# A Versatile Class of 1,4,4-Trisubstituted Piperidines Block Coronavirus Replication In Vitro

**DOI:** 10.3390/ph15081021

**Published:** 2022-08-18

**Authors:** Sonia De Castro, Annelies Stevaert, Miguel Maldonado, Adrien Delpal, Julie Vandeput, Benjamin Van Loy, Cecilia Eydoux, Jean-Claude Guillemot, Etienne Decroly, Federico Gago, Bruno Canard, Maria-Jose Camarasa, Sonsoles Velázquez, Lieve Naesens

**Affiliations:** 1 Instituto de Química Médica (IQM, CSIC), E-28006 Madrid, Spain; 2 Rega Institute for Medical Research, KU Leuven, B-3000 Leuven, Belgium; 3 AFMB, UMR 7257, CNRS, Aix Marseille Université, 13288 Marseille, France; 4 Unidad Asociada al IQM-CSIC, Área de Farmacología, Departamento de Ciencias Biomédicas, Universidad de Alcalá, E-28805 Alcalá de Henares, Spain

**Keywords:** 1,4,4-trisubstituted piperidine, Ugi reaction, antiviral compound, coronavirus, SARS-CoV-2, main protease

## Abstract

There is a clear need for novel antiviral concepts to control SARS-CoV-2 infection. Based on the promising anti-coronavirus activity observed for a class of 1,4,4-trisubstituted piperidines, we here conducted a detailed analysis of the structure–activity relationship of these structurally unique inhibitors. Despite the presence of five points of diversity, the synthesis of an extensive series of analogues was readily achieved by Ugi four-component reaction from commercially available reagents. After evaluating 63 analogues against human coronavirus 229E, four of the best molecules were selected and shown to have micromolar activity against SARS-CoV-2. Since the action point was situated post virus entry and lying at the stage of viral polyprotein processing and the start of RNA synthesis, enzymatic assays were performed with CoV proteins involved in these processes. While no inhibition was observed for SARS-CoV-2 nsp12-nsp7-nsp8 polymerase, nsp14 N7-methyltransferase and nsp16/nsp10 2’-O-methyltransferase, nor the nsp3 papain-like protease, the compounds clearly inhibited the nsp5 main protease (M^pro^). Although the inhibitory activity was quite modest, the plausibility of binding to the catalytic site of M^pro^ was established by in silico studies. Therefore, the 1,4,4-trisubstituted piperidines appear to represent a novel class of non-covalent CoV M^pro^ inhibitors that warrants further optimization and development.

## 1. Introduction

**Figure 1:**
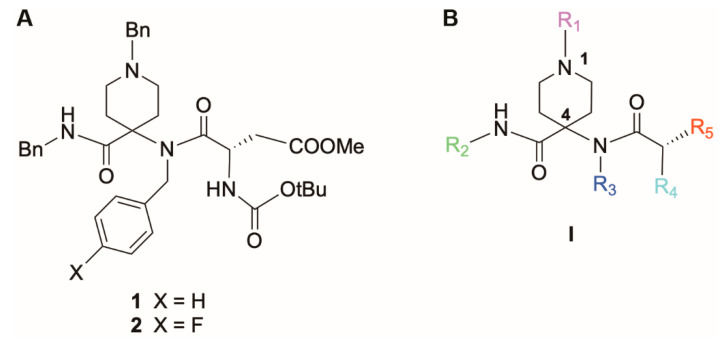
(**A**) chemical structure of compounds **1** and **2**, the two *N*-benzyl 4,4-disubstituted piperidines which we previously identified as inhibitors of influenza virus membrane fusion [10]. (**B**) general structure (**I**) of the library of 1,4,4-trisubstituted piperidine compounds that we synthesized in the present work to explore the SAR for anti-CoV activity.

Piperidine moiety is an important heterocycle for drug design since highly functionalized piperidines can exhibit diverse pharmacological or biological activities [1,2]. To prepare these compounds, different multicomponent reaction (MCR) strategies have been developed [3]. Currently, MCRs are an efficient and powerful synthetic tool to generate complex chemical libraries given that the products are formed from three or more reagents in one single step [4,5]. The Ugi-4CR (four-component reaction) is a versatile MCR for the expedient synthesis of α-aminoacyl amide derivatives with a wide variety of substitution patterns [6,7,8]. This makes this methodology very useful when generating compound libraries for screening purposes. Our group recently applied the highly efficient one-step Ugi-4CR to obtain a structurally diverse library of dipeptide derivatives bearing an *N*-benzylpiperidine scaffold [9,10]. When we evaluated these 1,4,4-trisubstituted piperidines for antiviral activity, several compounds showed low μM anti-influenza A/H1N1 virus activity [10]. Starting from hit **1**, the fluorinated analogue **2** (Figure 1) emerged as the most potent inhibitor. Based on selection of resistant virus and other mechanistic assays, we demonstrated that **2** represents a new class of inhibitors of the H1 hemagglutinin-mediated membrane fusion process [10].

Over the course of the above study focused on influenza virus, the 1,4,4-trisubstituted piperidines were also evaluated against several other viruses. Intriguingly, promising cell culture activity was observed for human coronavirus 229E (HCoV-229E), an endemic coronavirus (CoV) that causes relatively benign infections of the upper respiratory tract [11,12]. This finding gained full relevance after the emergence of SARS-CoV-2 and the renewed interest in CoV inhibitors that is fueled by the devastating COVID-19 pandemic [13,14]. There is a particular need for novel anti-CoV agents with a different mechanism than the drugs that are formally approved or in advanced clinical development, i.e., the covalent main protease (M^pro^) inhibitor nirmatrelvir [15,16,17]; polymerase inhibitors remdesivir [18,19,20], molnupiravir [21,22], favipiravir [23,24] and bemnifosbuvir (AT-527) [25,26]; and anti-spike antibodies [27,28]. Pan-coronavirus inhibitors are preferred since these will be applicable for any new coronavirus outbreak that may occur in the future [29].

In the study reported herein, we performed a detailed structure–activity relationship (SAR) analysis of these 1,4,4-trisubstituted piperidine-based inhibitors of CoV. MCR synthesis of an extensive series of analogues (see general structure **I** in Figure 1) allowed the structural elements that are essential for anti-CoV activity and selectivity to be defined. After evaluating the entire series against HCoV-229E, four of the best molecules were confirmed to be equally active against SARS-CoV-2. A time-of-compound addition experiment indicated that the action point of these inhibitors coincides with the stage of CoV polyprotein processing and the start of viral RNA synthesis [30]. Hence, we performed inhibition studies with the SARS-CoV-2 M^pro^ enzyme responsible for cleavage of the pp1a and pp1ab polyproteins [31], as well as enzymes involved in CoV RNA synthesis [30,32], namely nsp14 N7-methyltransferase and nsp16/nsp10 2’-O-methyltransferase (two main enzymes of the CoV RNA capping machinery) [26,33] and RNA-dependent RNA polymerase (RdRp; nsp12 in complex with the nsp7 and nsp8 accessory proteins) [34,35]. We conclude that the 1,4,4-trisubstituted piperidines represent a structurally unique class of CoV inhibitors purportedly acting on the M^pro^ enzyme which merits further development.

## 2. Results

### 2.1. Chemical Synthesis

**Scheme 1:**
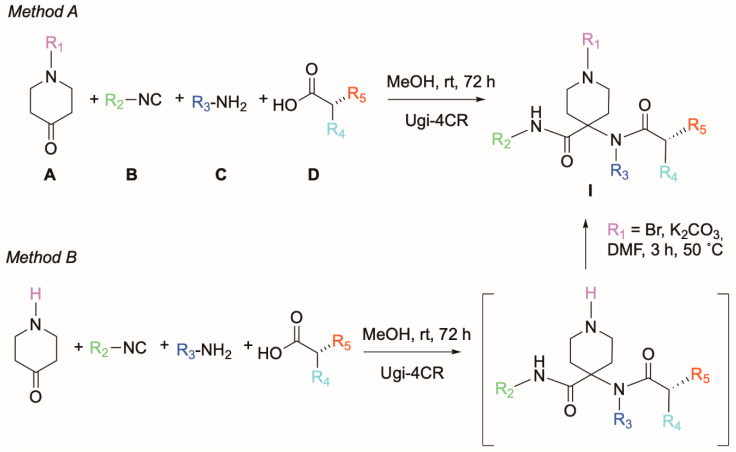
Synthesis of the novel piperidine analogues of general structure **I**, based on Ugi-4CR reaction.

We previously developed the one-step Ugi-4CR to prepare a structurally diverse library of 1,4,4-trisubstituted piperidines [10]. During broad antiviral testing, we observed that this compound class not only encompassed selective replication inhibitors of influenza virus (compounds **1** and **2** in Figure 1), but also of CoV (see below). While our first synthetic work was focused on influenza virus [10], we next decided to fully explore anti-CoV potential by synthesizing additional 1,4,4-trisubstituted piperidine analogues modified at substituents R_1_ to R_5_. The general synthetic routes are depicted in Scheme 1. Single step synthesis based on Ugi-4CR reaction yielded the target compounds in moderate to good yields. Thus, reaction of commercially available *N*-substituted 4-piperidone (A), isocyanides (B), primary amines (C) and a variety of polar, hydrophobic or aromatic natural (L) amino acids as carboxylic acids (D) in methanol at room temperature over 72 h (Method A) was performed. On the other hand, if the *N*-substituted 4-piperidone with the desired R_1_ modification was not commercially available, the Ugi-4CR reaction was performed with 4-piperidone (Method B) followed by *N*-piperidine alkylation of the Ugi reaction product with the corresponding R_1_-Br in the presence of K_2_CO_3_ in dry DMF at 50 °C for 3 h (Scheme 1). In Table 1, we provide the chemical structures of the piperidine analogues which were newly synthesized in the present study, i.e., **15, 16, 49**–**53** and **57**–**59** (Method A) and **54**–**56** and **60**–**63** (Method B).

### 2.2. Biological Activity

#### 2.2.1. SAR Analysis for Antiviral Activity against HCoV-229E 

When we evaluated **1** and **2** (both active against influenza virus [10]) in HCoV-229E-infected human embryonic lung (HEL) fibroblasts, **2** had an antiviral EC_50_ value of 7.4 μM while no activity was observed for its close analogue **1**, which lacks the 4-fluorine substituent in the R_3_-benzyl group (Table 1). With a CC_50_ (=50% cytotoxic concentration) value of 44 µM, **2** emerged as a poorly selective inhibitor with a selectivity index (= ratio of CC_50_ to EC_50_) of 6. Hence, the purpose of our SAR study was to increase both potency and selectivity. By combining our previously and newly synthesized compound series, we collected a library of 63 analogues representing a variety of 1,4,4-trisubstituted piperidines with modifications at R_1_ to R_5_. This diversity allowed us to perform an in-depth analysis of the SAR for CoV (Table 1). Since HCoV-229E produces robust cytopathic effect (CPE) in HEL cells that is quantifiable by microscopic scoring and MTS cell viability assay, we decided to use this model as the primary assay. 

In subseries 1, we addressed the contribution of the *N*-benzylpiperidine moiety (R_1_ substituent at the piperidine nitrogen). Antiviral activity was reduced when the benzyl substituent in **2** was replaced by a hydrogen, methyl, cyclohexyl or phenyl (**4**–**7**). Quaternization of this N atom by alkylation with MeI was detrimental (**8**). A negative impact was also seen when the *N*-benzyl moiety carried a fluorine at position 3 (**9**) or at both positions 3 and 5 (**10**). On the other hand, when the *N*-benzyl of **2** was replaced by *N*-phenylethyl (**11**), the anti-HCoV-229E activity was increased two-fold.

In subseries 2, we evaluated compounds modified at the R_2_ substituent, which we obtained by varying the isocyanides in the Ugi reaction. Here, a benzyl group proved superior to *tert*-butyl, cyclohexyl or tosyl in comparison to the fluorine prototype **2** (**12**–**14** vs. **2**). 

Subseries 3, modified at the R_3_ and/or R_4_ substituent, was achieved by varying the primary amine and/or the *N*-protecting group in the natural (*L)* Asp(OMe) amino acid that was employed to provide the carboxylic acid component in the Ugi reaction. Removal of the R_3_ benzyl group (**18**) or substitution by a (cyclo)alkyl group (**19**–**21**) was detrimental. On the other hand, replacing the 4-F in **2** with a 4-methyl (**22**) or 4-nitro (**23**) substituent, or substituting a PhNH(CH_2_)_2_ group for its R_3_ benzyl group (**29**), increased antiviral activity and selectivity by about two-fold. 4-CF_3_ (**24**) or 4-chlorine (**25**) retained anti-CoV activity but increased cytotoxicity (**24**–**28**). Changing the 4-fluorine to position 2 (**26**) or position 3 (**27**) did not have any effects on either activity or toxicity, while the 3,4-F-disubstituted derivative (**28**) was more cytotoxic. Regarding the R_4_ substituent, removal of the *N*-protecting group caused loss of activity or higher toxicity (**30**–**32** vs. **2**). Importantly, exchange of the NH-Boc by an NH-Cbz group (**33** vs. **1** and **34** vs. **2**) was highly beneficial as it increased antiviral potency and, even more so, the selectivity index. With an EC_50_ value of 3.3 µM and no visible cytotoxicity at 100 µM (the highest concentration tested), compound **33** proved to be the strongest inhibitor within the entire series. This impact of the NH-Cbz is quite remarkable, considering that its NH-Boc counterpart, compound **1**, was inactive. Using other aromatic *N*-protecting groups (NH-Fmoc) or combining the NH-Cbz with an R_3_ alkyl (instead of the original benzyl) substituent led to a clear reduction in antiviral activity or selectivity (compounds **35**–**37** vs**. 33**).

Regarding modifications at R_5_ (subseries 4), elimination, replacement by Me, chain elongation or substitution of COOMe by CONH_2_ reduced anti-CoV potency (**38**–**42**), whereas replacement of COOMe by COOH (**43**, **44**) led to inactive compounds. Interestingly, substitution by a benzyl group at R_5_ was beneficial for activity. However, the concomitant presence at R_3_ of 4-F-Bn increased toxicity, while R_3_-benzyl decreased toxicity (**45** vs**. 46**). Based on these results, five derivatives of the benzyl-substituted compound **46** were synthesized, bearing different electron-donating or electron-withdrawing groups. Compared to the unsubstituted molecule **46**, the 4-OH, 4-F and 4-Cl analogues (**48**–**50**) were less active by two-fold. The 4-OMe (**51**) and 4-Me (**52**) substituted analogues were as active as **46** but less cytotoxic (CC_50_ > 100 µM; SI > 19).

Finally, in subseries 5, we combined the most promising elements at R_1_-R_5_ in the hope that this would maximize antiviral activity. Combining an (un)substituted phenylethyl group at R_1_ with the favorable NHCbz at R_4_ proved, unfortunately, to be neither beneficial for activity nor toxicity (**53**–**56** vs. **11** and **33**). Similarly, when the best substituents at R_3_ [PhNH(CH_2_)_2_ or 4-Me-Bn] were combined with phenylethyl at R_1_ or NHCbz at R_4_, the new compounds were less active and selective than the parent compounds (**57**–**59** vs**. 11**, **29** and **33**). Finally, we introduced substituents at the R_1_ benzyl group of **46**, one of the more selective analogues. The 4-OMe derivative was more cytotoxic (**62** vs. **46**) and the 4-Cl and 4-NO_2_ analogues proved markedly less active (**61** and **63** vs**. 46**), while the 4-F substituent yielded a highly potent and less cytotoxic compound (**60**) with an SI > 22.

#### 2.2.2. Inhibitory Activity against SARS-CoV-2

After gaining thorough insight into the SAR for HCoV-229E, we evaluated four of the best analogues for their ability to inhibit replication of SARS-CoV-2 (clinical isolate of early 2020). As a suitable cell model, we chose human lung carcinoma A549 cells stably transfected with human ACE2 and TMPRSS2 (abbreviated: A549-AT). These host cell factors function, respectively, as a receptor and as an activating protease for spike protein-mediated entry of SARS-CoV-2 [37]. A549-AT cells are a relevant model for testing compounds against SARS-CoV-2, and preferable to Vero cells in which efflux mechanisms may reduce the activity of test compounds [38]. To monitor antiviral activity in a direct manner, we performed an RT-qPCR-based viral load reduction assay in which the viral RNA copy number in the supernatant was determined at day 3 p.i. [39]. In parallel, mock-infected cultures were exposed to the compounds to assess their cytotoxicity using an MTS cell viability assay. 

**Figure 2:**
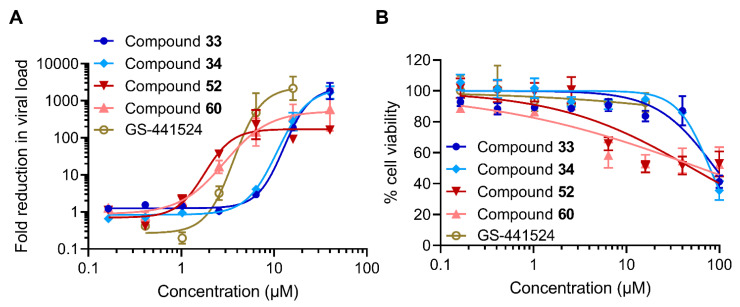
Inhibition of SARS-CoV-2 replication in A549-AT cells. (**A**) reduction in viral load in the supernatant at day 3 p.i. (**B**) compound cytotoxicity based on MTS cell viability assay in mock-infected cells. Data points are the mean ± SEM (N = 3). Reference compound: GS-441524, the nucleoside form of remdesivir.

As evident from the dose–response curves in Figure 2, the four piperidine compounds generated 2- to 3-log_10_ reductions in viral load, which are comparable to the reduction obtained with GS-441524, the nucleoside form of remdesivir. Compound **52**, a 4-Me substituted Phe analogue at R_5_, exhibited the lowest EC_90_ and EC_99_ values (Table 2), followed by **60,** a 4-F-Bn derivative substituted at R_1_. Their EC_99_ values for SARS-CoV-2 (3.9 µM for **52** and 5.2 µM for **60**) were practically identical to the EC_50_ values noted in the CPE assay with HCoV-229E (Table 1, EC_50_-MTS: 4.7 µM for **52** and 4.0 µM for **60**). Likewise, **33** and **34** proved almost identical in terms of anti-SARS-CoV-2 potency and selectivity, indicating that the 4-F substituent on the R_3_ benzyl group has little influence. This finding conflicts with our initial hypothesis, which was based on parent compounds **1** and **2**, but aligns with the elaborate SAR findings for HCoV-229E, in which several non-fluorinated R_3_-benzyl analogues (for example: **22**–**25**, **29**, **46**, **51** or **53**) exhibited notable activity.

#### 2.2.3. Time-of-Compound Addition Profile 

**Figure 3:**
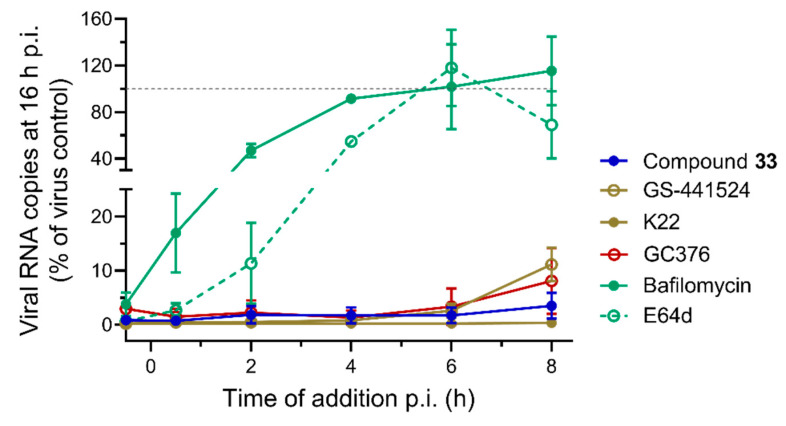
One-cycle TOA experiment in HCoV-229E-infected HEL cells. Compounds and concentrations: 1,4,4-trisubstituted piperidine analogue **33** at 15 µM; inhibitors of virus entry: E64d (15 µM) and bafilomycin (0.00625 µM); M^pro^ inhibitor GC376 (25 µM); and inhibitors of viral RNA synthesis: GS-441524 (20 µM) and K22 (15 µM). Data points are the mean ± SEM (N = 3 for **33** and N = 2 for the reference compounds). The cells were exposed to the compounds at the indicated time points, infected at time point zero, and lysed at 16 h p.i. The Y-axis shows the number of intracellular viral RNA copies, determined by RT-qPCR and expressed relative to the value in the virus control (=set at 100%; dashed grey line).

To gain further insight into the antiviral mechanism, we conducted a one-cycle time-of-compound addition (TOA) experiment in HCoV-229E-infected HEL cells, in which **33** was compared to five reference compounds (Figure 3). E64d and bafilomycin, two inhibitors of HCoV-229E entry [40] which act on endosomal cathepsins and V-ATPases, respectively, provided only 46% (E64d) and 9% (bafilomycin) inhibition of viral RNA synthesis when their addition was delayed until 4 h p.i. The CoV M^pro^ inhibitor GC376 [41,42] and polymerase inhibitor GS-441524 [43] started to lose activity when added at 8 h p.i. but still produced ~90% inhibition of viral RNA synthesis when added at this time point. The similar time profile for these two compounds suggests that viral RNA synthesis starts shortly after M^pro^-mediated polyprotein cleavage, as also seen in a TOA study with SARS-CoV-2 [38]. Compound **33** resembled GC376 and GS-441524 in losing some activity when added at 8 h p.i., while the reference molecule K22 remained 100% effective. It has been proposed that K22 acts on nsp6, a CoV non-structural protein that is implicated in the formation of double-membrane vesicles in which viral genome replication and transcription take place [36,44]. Hence, **33** acts at a replication stage occurring after virus entry and coinciding with polyprotein processing and the start of viral RNA synthesis. Based on the finding that **33** potently suppresses the expansion of viral RNA within the first cycle, we can exclude a late effect during virus maturation or release, which occurs after RNA synthesis [30].

#### 2.2.4. Evaluation against Enzymes of the SARS-CoV-2 Replication–Transcription Complex and the Two Viral Proteases 

To further decipher the mode of action, we first evaluated a selection of the 1,4,4-trisubstituted piperidine compounds against key enzymes of the SARS-CoV-2 replication–transcription complex (RTC), the protein assembly responsible for viral RNA synthesis [30]. Using a recently developed fluorescence-based assay to monitor RNA synthesis by the RdRp (i.e., nsp12 in complex with nsp7 and nsp8) [35], we did not detect any significant inhibition by compounds **1**, **33**, **34**, **46**, **52**, **60** or **63** (highest concentration tested: 100 µM). Next, we tested the compounds against the N7- and 2’O-methyltransferase (MTase) enzymes involved in cap structure methylation using radioactive enzymatic assays [33,45]. The nsp14 N7-MTase or nsp10/nsp16 protein complex responsible for 2’O-methylation of the first nucleotide of the cap structure was incubated for 10 min with different concentrations of **33**, **34**, **52** and **60**. Thereafter, the enzymatic activity of the two MTases was measured by filter-binding assay. The compounds had either no effect or only a marginal effect, giving at best 80% inhibition with compound **34** (nsp14 N7-MTase) and compound **60** (nsp10/nsp16 2’O-MTase) at a compound concentration of 200 µM. Hence, we can exclude direct inhibition of SARS-CoV-2 RdRp, nsp14 N7-MTase or nsp10/nsp16 2’O-MTase as the mechanistic basis for the anti-CoV activity in cell cultures. 

The TOA data suggested potential interference with the processing of polyproteins pp1a or pp1ab by the nsp3 papain-like protease (PL^pro^) and nsp5 main protease (M^pro^). Because of its distant resemblance to picornavirus 3C proteases, M^pro^ is also referred to as 3C-like proteinase (3CL^pro^) [46]. Hence, we tested the compounds against SARS-CoV-2 nsp3 using a fluorogenic assay with Cbz-RLRGG-AMC substrate, yet no inhibition was observed. On the other hand, nsp5 M^pro^ activity was clearly inhibited since the compounds reduced the increase in fluorescence when incubated with nsp5 protein and a synthetic peptide mimicking the nsp5 cleavage site. The IC_50_ values calculated for the eight tested compounds are presented in Table 3. Whereas compounds **45**, **46** and **52** had the best potency (IC_50_ values between 14 and 22 µM), compounds **33**, **34** and **60** had an IC_50_ value between 38 and 68 µM, and the other two (**1** and **63**) had only marginal activity. Although these high IC_50_ values do not argue for strong inhibition, the effect towards M^pro^ might be potentially amplified in infected cells and/or combined with another so far unknown mechanism.

Serial dilutions of the compounds were incubated with purified nsp5 protein in the presence of substrate (Dabcyl-KTSAVLQ↓SGFRKM-Edans-NH_2_) [47], and peptide cleavage was followed by an increase in fluorescence. The compounds’ IC_50_ values were determined by Hill curve fitting. Values are the mean ± SEM (N = 3). 

The observed inhibition of M^pro^ led us to evaluate the compounds against cellular proteases with a known role in CoV entry or maturation, even though the TOA results did not favor an action point during these replicative stages (see above). Hence, we tested the compounds against: furin (involved in CoV spike cleavage during virus assembly or budding), trypsin (an analogue of TMPRSS2, a protease associated with virus entry at the cell surface) and cathepsin B and cathepsin L (which activate the spike after virus entry via endocytosis) [39,48]. We used commercially available enzymes and peptide substrates, as well as similar fluorogenic assays as used for nsp3 and nsp5. None of the 1,4,4-trisubstituted piperidines tested inhibited the enzymatic activity of human furin, trypsin, cathepsin B or cathepsin L (highest concentration tested: 100 µM). Thus, none of these proteases seem to be the target behind the anti-CoV effect or compound cytotoxicity at higher concentrations.

### 2.3. In Silico Modeling

**Figure 4:**
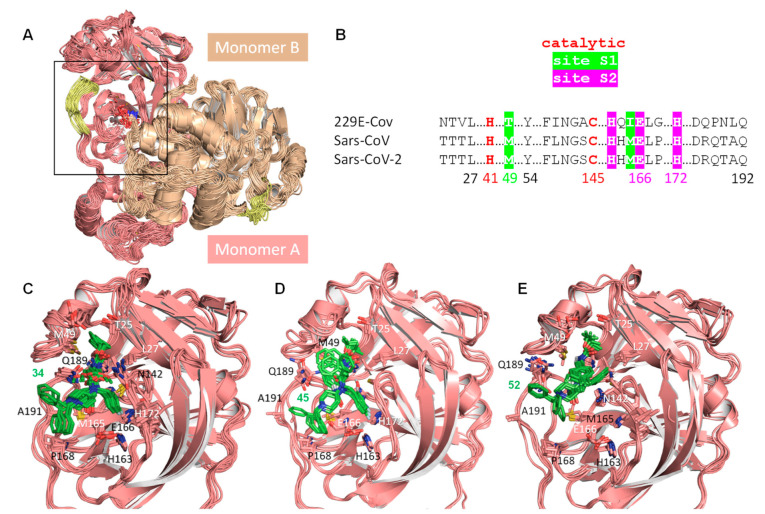
Ribbon representation of chymotrypsin-like protease M^pro^ from SARS-CoV-2. (**A**) Overlay of 30 snapshots taken at regular intervals from the unrestrained molecular dynamics simulation in water lasting 150 ns. Each monomer is displayed in a different color and the ^187^Asp-Ala^193^ stretch is highlighted in yellow. (**B**) Alignment of active site amino acids from HCoV-229E, SARS-CoV and SARS-CoV-2 M^pro^ enzymes that are proposed to have a direct bearing on ligand binding, as discussed in the text. (**C**–**E**) Theoretical models of one monomer of SARS-CoV-2 M^pro^ (ribbon with C atoms colored in pink) in complex with compound **34** (**C**), **45** (**D**) and **52** (**E**); the region displayed corresponds to the boxed area in (**A**). Ligands are shown as sticks with carbon atoms colored in green. Each set of five superposed structures represents a conformational ensemble made up of snapshots taken every 5 ns from the post-equilibrated 75–100 ns interval of the simulated trajectories and then cooled down to 273 K and energy minimized. For reference, some of the residues closest to the ligands have been labeled. Water molecules are not displayed for enhanced clarity.

SARS-CoV-2 M^pro^ was simulated as the biologically relevant dimer [49] and as a monomer, both in the apo form and in complex with different inhibitors. The top-scoring poses, generated by the automated docking program in the active site and thereafter subjected to molecular dynamics (MD) simulations for 100 ns, showed a notable convergence for inhibitors **34**, **45** and **52**. The equilibrated complexes showed that the ligands remain in the binding cavity (Figure 4) and adapt their shape and functional groups in a similar but subtly different way, depending on whether the amino protecting group at R_4_, which is lodged in site S2, is either a *tert*-butylcarbamate or a benzylcarbamate, and the R_5_ substituent contains a methylcarboxylate or a phenyl ring. Direct yet transient hydrogen bonds are only apparent with the backbone NH of Glu166 transiently, although the scaffold’s C=O and NH groups can establish additional water-mediated hydrogen bonds with both backbone polar atoms and the side chains of Asn142 and Gln189, which together sandwich the bound inhibitor. The benzyl at R_1_ displays considerable conformational freedom but is seen to interact with the side chains of Pro168 and Ala191 in site S4. The benzyl substituent at R_2_ gets close to the side-chain thioether of Met165. The fluorobenzyl moiety at R_3_ appears to promote insertion of this moiety into the crevice lined by Ser144 and Glu166 (site S1) and establishment of hydrogen-bonding interactions between the fluorine atom—when present—and the imidazole ring of His163. When hydrogen occupies the same *para* position, the benzyl moiety appears to adopt a different conformation that is translated into a slight repositioning of the inhibitor within the active site.

## 3. Discussion

This study started from the serendipitous observation that the 1,4,4-trisubstituted piperidine **2** exhibited promising antiviral activity in a CPE reduction assay with HCoV-229E. Since we previously identified this molecule as an inhibitor of influenza virus H1 hemagglutinin-mediated membrane fusion during virus entry, we initially assumed that its anti-CoV activity might be related to inhibition of CoV entry. This possibility is definitely excluded by our TOA experiment with the selective analogue **33**, the outcome of which pointed to inhibition at the stage of CoV polyprotein cleavage or the start of viral RNA synthesis. Our enzymatic findings do not support that the compounds directly inhibit the nsp14 and nsp10/16 methyltransferases or nsp12/nsp7/nsp8 polymerase. In contrast, consistent with their peptidomimetic nature, the compounds do inhibit the enzymatic activity of nsp5 M^pro^, a key player in CoV polyprotein cleavage and a well-established antiviral drug target [15,31,49,50,51]. Although the relatively high IC_50_ values of our inhibitors may cast doubts on the relevance of these enzymatic findings to account for the antiviral activity observed in infected cells, the likelihood of their interaction with M^pro^ was assessed by in silico studies. Specifically, the results from our molecular modeling studies hint at a plausible binding mode that entails tight fitting of the inhibitor into the active site of this enzyme by virtue of a high degree of steric complementarity and formation of direct and water-mediated hydrogen bonds with the backbone of Glu166 as well as the side chains of Asn142 and Gln189. It is conceivable that these results can be safely extrapolated to the highly homologous M^pro^ enzymes from SARS-CoV and HCoV-229E (Figure 4) [50,52].

Definite validation that the 1,4,4-trisubstituted piperidine analogues act by targeting M^pro^ awaits independent methods, particularly selection of resistant CoV mutants. So far, we have not succeeded in obtaining HCoV-229E resistant mutants when using either a traditional approach of serial virus passaging or a faster method with brief exposure to high viral multiplicity of infection and high compound concentration. This indicates that the resistance barrier of these CoV inhibitors must be quite high. For SARS-CoV-2, resistance studies are hindered by the rather limited window between antiviral activity and cytotoxicity in A549-AT cells. Apart from this restriction, the substituted piperidine analogues generated up to 1000-fold reduction in SARS-CoV-2 viral load, which was comparable to the inhibition seen with the nucleoside form of remdesivir.

**Figure 5:**
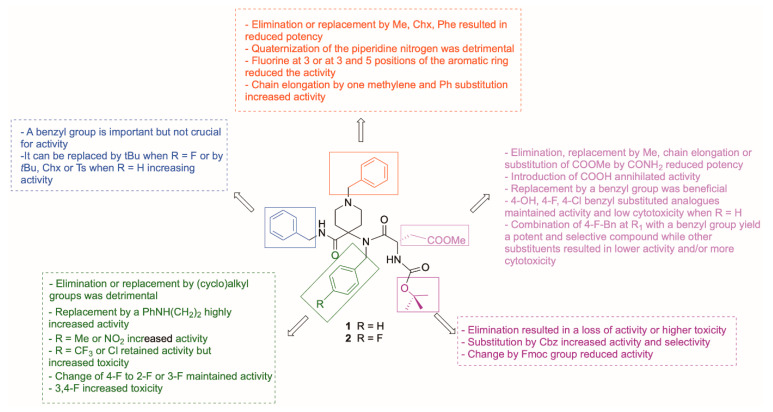
Current insights related to the SAR based on evaluation of compounds **1**–**63** against HCoV-229E.

To conclude, the five points of diversity make these N-substituted piperidine-based compounds highly versatile and amenable to further optimization. In Figure 5, we summarized the insights gained in relation to the SAR from the 63 analogues that we have synthesized so far. These compounds represent a novel, structurally unique and versatile class of CoV inhibitors. The combined results from TOA and enzymatic assays, and in silico molecular modeling, lend credence to our proposal that they inhibit M^pro^ via a non-covalent mechanism, making these inhibitors fundamentally different from other M^pro^ inhibitors represented by the approved drug nirmatrelvir [53] and others [54,55,56]. Therefore, these CoV inhibitors warrant further investigation related to maximizing their activity and selectivity and gaining full insight into their antiviral mechanism.

## 4. Materials and Methods

### 4.1. Chemical Synthesis

#### 4.1.1. Instrumentation and Chemicals

A Heraeus CHN-O-RAPID was used to perform microanalyses and the results corresponded to the theoretical values ± 0.4%. Liquid chromatography–mass spectrometry was carried out on a quadrupole mass spectrometer equipped with an electrospray source (HP 1100, Hewlett Packard). Silica gel 60 F254 (Merck) was used for analytical thin-layer chromatography. The compounds were purified by flash column chromatography (silica gel 60, 230-400 mesh, Merck) on a Chromatotron (Kiesegel 60 PF254 gipshaltig, Merck) by preparative centrifugal circular thin-layer chromatography (1 mm layer, flow rate 5 mL/min), or by medium pressure liquid chromatography on an Isolera One system using SNAP 12 g kP-C18-HS cartridges (Biotage). Next, their purity was determined by analytical reversed-phase HPLC on an Agilent 1120 Compact LC instrument with an ACE 5 C18-300 column (150 mm × 4.6 mm) and a mobile phase composed of acetonitrile/water with 0.05% TFA (gradient; flow rate of 1 mL/min; UV detection at 217 nm). Retention times are expressed in minutes. HPLC-MS analysis was conducted on a Waters 2695 HPLC equipped with a Sunfire C18 column (4.6 mm × 50 mm, 3.5 mm; flow rate: 1 mL/min) connected to a Micromass ZQ 2000 spectrometer (Waters) and a photodiode array detector. NMR spectra were recorded on a Varian Inova 400 or Varian System 500 spectrometer (at 400 or 500 MHz for ^1^H NMR and at 100 or 125 MHz for ^13^C NMR) using tetramethylsilane as an internal standard. The purity of the compounds was >95%, as also measured by elemental analysis. Chemicals and reagents from commercial sources were used without further purification.

#### 4.1.2. General Procedure for the Ugi Reaction (Method A)

The ketone (1.32 mmol) was dissolved in methanol (2 mL) and the amine, amino acid and isocyanide were successively added (2 equivalents of each) to this solution. After 4 days at room temperature with stirring, 1.2 M hydrochloric acid in methanol was added, and the mixture was further stirred for an additional 30 minutes at room temperature. After removal of the solvents, the residue was treated with ethyl acetate, washed with saturated sodium bicarbonate (3 × 10 mL), then washed with brine (3 × 10 mL). The organic phase was filtered after drying with magnesium sulphate and finally evaporated to dryness. Flash column chromatography (hexane:ethyl acetate, 4:1 to 0:1) was used to purify the final residue to yield the 1,4,4-trisubstituted piperidine analogues **15, 16, 49**–**53** and **57**–**59**. Details are given below for each compound.

Methyl(S)-4-(benzyl(1-benzyl-4-*tert*-butylcarbamoylpiperidin-4-yl)amino)-3-*tert*-butoxycarbonylamino-4-oxobutanoate (**15**): Reaction of 1-benzylpiperidin-4-one (0.22 mmol, 0.016 mL), benzylamine (0.44 mmol, 0.071 mL), Boc-Asp(OMe)-OH (0.44 mmol, 122 mg) and *tert*-butyl isocyanide (0.44 mmol, 0.066 mL) in MeOH (2 mL) yielded **15** (98 mg, 73%) as a white foam. ^1^H NMR (500 MHz, DMSO-d6) δ 7.51 (d, *J* = 7.6 Hz, 2H), 7.36 (t, *J* = 7.6 Hz, 3H), 7.25 (m, 3H), 7.18 (m, 3H), 6.17 (s, 1H), 4.79 (d, *J* = 18.1 Hz, 1H), 4.69 (q, *J* = 7.0 Hz, 1H), 4.62 (d, *J* = 18.1 Hz, 1H), 3.56 (s, 3H), 3.43 (m, 1H), 2.84 (dd, *J* = 16.4, 6.8 Hz, 1H), 2.50 (m, 1H), 2.41 (m, 4H), 2.26–2.12 (m, 2H), 1.98 (d, *J* = 10.9 Hz, 1H), 1.81 (m, 1H), 1.58 (m, 1H), 1.30 (s, 9H), 1.16 (s, 9H). ^13^C NMR (126 MHz, DMSO-d6) δ 171.9, 171.6, 171.4, 155.5, 139.6, 138.9, 129.1, 128.6, 128.5, 127.6, 127.2, 78.9, 78.8, 64.7, 62.3, 55.3, 51.9, 50.3, 49.6, 47.4, 36.5, 31.0, 28.6, 28.5. MS (ES+) *m/z*: 609.49 (M + 1)^+^. HPLC retention time = 8.31 min (96% analytical purity) (H_2_O/CH_3_CN from 15/85 to 0/100 in 5 min flow rate of 1 mL/min). Anal. calculated for C_34_H_48_N_4_O_6_ (C, H, N): C, 67.08; H, 7.95; N, 9.20. Found: C, 67.32; H, 7.84; N, 9.53.

Methyl(S)-4-(benzyl(1-benzyl-4-cyclohexylcarbamoylpiperidin-4-yl)amino)-3-*tert*-butoxycarbonylamino-4-oxobutanoate (**16**): Following the general procedure, a solution of 1-benzylpiperidin-4-one (0.22 mmol, 0.016 mL), benzylamine (0.44 mmol, 0.071 mL), Boc-Asp(OMe)OH (0.44 mmol, 122 mg) and cyclohexyl isocyanide (0.44 mmol, 0.066 mL) in MeOH (2 mL) was reacted. Compound **16** (81 mg, 58%) was obtained as a white foam after purification. ^1^H NMR (500 MHz, DMSO-d6) δ 7.46 (d, *J* = 7.6 Hz, 2H), 7.36 (t, *J* = 7.6 Hz, 2H), 7.27 (m, 1H), 7.23 (dd, *J* = 8.0, 6.5 Hz, 3H), 7.17 (m, 1H), 7.13 (m, 2H), 6.77 (d, *J* = 8.0 Hz, 1H), 4.76 (d, *J* = 18.4 Hz, 1H), 4.68 (m, 2H), 3.55 (s, 3H), 3.42 (m, 1H), 3.26 (d, *J* = 5.6 Hz, 2H), 3.16 (d, *J* = 5.1 Hz, 1H), 2.84 (dd, *J* = 16.5, 5.6 Hz, 1H), 2.45 (m, 2H), 2.39 (m, 2H), 2.31 (m, 1H), 2.15 (m, 2H), 1.78 (td, *J* = 12.3, 4.1 Hz, 1H), 1.62 (m, *J* = 9.2, 4.2 Hz, 4H), 1.52 (m, 2H), 1.29 (s, 9H), 1.19 (m, 2H), 1.09 (m, 2H). ^13^C NMR (126 MHz, DMSO-d6) δ 171.9, 171.4, 171.4, 155.5, 139.2, 138.9, 129.2, 129.1, 128.9, 128.5, 127.5, 127.3, 127.2, 78.8, 64.1, 62.2, 51.9, 50.3, 49.9, 49.5, 47.9, 47.8, 36.6, 32.8, 32.6, 32.1, 28.5, 25.8, 25.2. MS (ES+) *m/z* 635.59 (M + 1)^+^. HPLC retention time = 8.53 min (99% analytical purity) (H_2_O/CH_3_CN from 15/85 to 0/100 in 5 min flow rate of 1 mL/min). Anal. calculated for C_36_H_50_N_4_O_6_ (C, H, N): C, 68.11; H, 7.94; N, 8.83. Found: C, 68.33; H, 7.76; N, 8.64.

*Tert*-butyl(S)-(1-(benzyl(1-benzyl-4-benzylcarbamoylpiperidin-4-yl)amino)-3-(4-fluorophenyl)-1-oxopropan-2-yl)carbamate (**49**): Following the general procedure, a solution of 1-benzylpiperidin-4-one (0.22 mmol, 0.016 mL), benzylamine (0.44 mmol, 0.071 mL), Boc-4-fluorophenylalanine (0.44 mmol, 122 mg) and benzyl isocyanide (0.44 mmol, 0.066 mL) in MeOH (2 mL) was reacted. After purification of the final residue, compound **49** (109 mg, 73%) was obtained as a white foam. ^1^H NMR (500 MHz, DMSO-d6) δ 7.84 (t, *J* = 6.0 Hz, 1H), 7.35 (m, 17H), 7.02 (m, 2H), 6.98 (m, 1H), 5.84 (m, 2H), 4.28 (m, 3H), 3.30 (m, 2H), 2.89 (dd, *J* = 14.0, 4.2 Hz, 1H), 2.68 (dd, *J* = 14.0, 9.8 Hz, 1H), 2.52 (m, 1H) 2.42 (m, 4H), 2.26 (m, 1H), 1.80 (m, 1H), 1.55 (m, 1H), 1.21 (s, 9H). ^13^C NMR (126 MHz, DMSO-d6) δ 173.6, 172.8, 161.4 (d, *J_C-F_* = 241.7 Hz), 155.8, 140.4, 139.5, 138.8, 134.6 (d, *J_C-F_* = 2.9 Hz), 131.5 (d, *J_C-F_* = 8.0 Hz), 129.3, 129.2, 129.07, 128.9, 128.8, 128.5, 128.5, 127.6, 127.5, 127.45, 127.3, 127.2, 126.9, 126.9, 126.6, 115.1 (d, *J_C-F_* = 21.0 Hz), 78.5, 63.9, 62.2, 55.5, 55.4, 54.5, 50.1, 49.8, 48.1, 42.8, 38.3, 37.0, 32.7, 32.2, 28.4, 27.7. MS (ES+) *m/z*: 679.5 (M + 1)^+^. HPLC retention time = 6.98 min (98% analytical purity) (H_2_O/CH_3_CN from 15/85 to 0/100 in 5 min flow rate of 1 mL/min). Anal. calculated for C_41_H_47_FN_4_O_4_ (C, H, F, N): C, 72.54; H, 6.98; F, 2.80; N, 8.25. Found: C, 72.26; H, 6.90; F, 2.91; N, 8.7.

*Tert*-butyl(1-(benzyl(1-benzyl-4-benzylcarbamoylpiperidin-4-yl)amino)-3-(4-chlorophenyl)-1-oxopropan-2-yl)carbamate (**50**): According to the general procedure, a solution of 1-benzylpiperidin-4-one (0.22 mmol, 0.016 mL), benzylamine (0.44 mmol, 0.071 mL), Boc-4-chlorophenylalanine (0.44 mmol, 122 mg) and benzyl isocyanide (0.44 mmol, 0.066 mL) in MeOH (2 mL) was reacted. Compound **50** (142 mg, 93%) was obtained as a white foam. ^1^H NMR (500 MHz, DMSO-d6) δ 7.85 (t, *J* = 6.0 Hz, 1H), 7.23 (m, 16H), 7.13 (d, *J* = 8.0 Hz, 2H), 7.02 (d, *J* = 8.0 Hz, 2H), 4.88 (d, *J* = 18.7 Hz, 1H), 4.79 (d, *J* = 18.7 Hz, 1H), 4.31 (m,1H), 4.26 (m,1H), 3.28 (s, 2H), 2.90 (dd, *J* = 13.9, 4.2 Hz, 1H), 2.67 (dd, *J* = 13.9, 9.8 Hz, 1H), 2.53 (m, 1H), 2.40 (m, 4H), 2.37 (m, 1H), 2.25 (t, *J* = 11.9 Hz, 1H), 1.79 (m, 1H), 1.53 (m, 1H), 1.20 (s, 9H). ^13^C NMR (126 MHz, DMSO-d6) δ 173.5, 172.9, 172.8, 172.6, 155.7, 140.4, 139.5, 138.8, 137.5, 131.7, 131.5, 131.4, 129.3, 129.2, 129.1, 128.9, 128.8, 128.5, 128.5, 128.4, 127.7, 127.5, 127.4, 127.2, 126.9, 126.9, 126.6, 78.6, 78.5, 63.9, 63.9, 62.2, 54.3, 50.1, 49.7, 48.1, 42.8, 37.2, 32.7, 32.2, 28.4. MS (ES+) *m/z*: 695.46 (M)^+^, 697.45 (M+2)^+^. HPLC retention time = 9.45 min (95% analytical purity) (H_2_O/CH_3_CN from 15/85 to 0/100 in 5 min flow rate of 1 mL/min). Anal. calculated for C_41_H_47_ClN_4_O_4_ (C, H, Cl, N): C, 70.83; H, 6.81; Cl, 5.10; N, 8.06. Found: C, 70.55; H, 6.93; Cl, 5.24; N, 8.34.

*Tert*-butyl(1-(benzyl(1-benzyl-4-benzylcarbamoylpiperidin-4-yl)amino)-3-(4-methoxyphenyl)-1-oxopropan-2-yl)carbamate (**51**): Reactants: 1-benzylpiperidin-4-one (0.22 mmol, 0.016 mL), benzylamine (0.44 mmol, 0.071 mL), Boc-4-methoxylphenylalanine (0.44 mmol, 122 mg) and benzyl isocyanide (0.44 mmol, 0.066 mL) in MeOH (2 mL). **51** was obtained (140 mg, 92%) as a white foam. ^1^H NMR (500 MHz, DMSO-d6) δ 7.81 (t, *J* = 6.0 Hz, 1H), 7.25 (m, 16H), 6.93 (d, *J* = 8.4 Hz, 2H),6.73 (d, *J* = 8.4 Hz, 2H), 4.91 (d, *J* = 18.5 Hz, 1H), 4.78 (d, *J* = 18.5 Hz, 1H), 4.28 (m, 3H), 3.66 (s, 3H), 3.28 (m, 3H), 2.85 (dd, *J* = 14.0, 4.1 Hz, 1H), 2.62 (dd, *J* = 14.0, 9.6 Hz, 1H), 2.52 (m, 1H), 2.40 (m, 3H), 2.26 (t, *J* = 11.5 Hz, 1H), 1.77 (m, 1H), 1.55 (bt, *J* = 9.5 Hz, 1H), 1.24 (s, 9H). ^13^C NMR (126 MHz, DMSO-d6) δ 173.9, 172.8, 158.2, 155.8, 140.4, 139.5, 138.9, 130.7, 130.3, 129.3, 129.2, 129.0, 129.0, 128.5, 128.5, 127.7, 127.6, 127.5, 127.4, 127.3, 127.2, 126.9, 126.9, 126.6, 113.9, 78.5, 78.4, 63.9, 62.2, 55.4, 54.8, 50.3, 50.1, 49.8, 48.1, 42.8, 36.9, 32.129, 28.5, 27.8, 19.1. MS (ES+) *m/z*: 691.57 (M + 1)^+^. HPLC retention time = 8.92 min (95% analytical purity) (H_2_O/CH_3_CN from 15/85 to 0/100 in 5 min flow rate of 1 mL/min). Anal. calculated for C_42_H_50_N_4_O_5_ (C, H, N): C, 73.02; H, 7.29; N, 8.11. Found: C, 73.22; H, 7.05; N, 8.38.

*Tert*-butyl(1-(benzyl(1-benzyl-4-benzylcarbamoylpiperidin-4-yl)amino)-1-oxo-3-(p-tolyl)propan-2-yl)carbamate (**52**): Reaction of 1-benzylpiperidin-4-one (0.22 mmol, 0.016 mL), benzylamine (0.44 mmol, 0.071 mL), Boc-4-methylphenylalanine (0.44 mmol, 122 mg) and benzyl isocyanide (0.44 mmol, 0.066 mL) in MeOH (2 mL) gave **52** (119 mg, 80%) as a white foam. ^1^H NMR (500 MHz, DMSO-d6) δ 7.82 (t, *J* = 6.0 Hz, 1H), 7.26 (m, 16H), 6.97 (d, *J* = 7.6 Hz, 2H), 6.89 (d, *J* = 7.6 Hz, 2H), 4.92 (d, *J* = 18.5 Hz, 1H), 4.78 (d, *J* = 18.5 Hz, 1H), 4.27 (m, 3H), 3.28 (s, 3H), 2.88 (dd, *J* = 14.0, 4.0 Hz, 1H), 2.64 (dd, *J* = 14.0, 9.6 Hz, 1H), 2.51 (m, 1H), 2.40 (m, 3H), 2.26 (t, *J* = 11.4 Hz, 1H), 2.20 (s, 3H), 1.79 (m, 1H), 1.55 (m, 1H), 1.24 (s, 9H). ^13^C NMR (126 MHz, DMSO-d6) δ 173.9, 173.3, 172.8, 172.6, 155.8, 153.8, 140.4, 139.5, 139.1, 138.9, 135.6, 135.6, 135.4, 129.7, 129.5, 129.3, 129.2, 129.0, 128.9, 128.5, 128.5, 127.6, 127.5, 127.4, 127.2, 126.9, 126.9, 126.57, 78.5, 78.4, 63.9, 62.2, 55.5, 54.7, 50.3, 50.1, 49.8, 48.1, 42.8, 38.9, 37.4, 32.9, 32.7, 32.2, 28.5, 27.8, 21.1. MS (ES+) *m/z*: 675.54 (M + 1)^+^. HPLC retention time = 9.35 min (99% analytical purity) (H_2_O/CH_3_CN from 15/85 to 0/100 in 5 min flow rate of 1 mL/min). Anal. calculated for C_42_H_50_N_4_O_4_ (C, H, N): C, 74.75; H, 7.47; N, 8.30. Found: C, 74.91; H, 7.21; N, 8.63.

Methyl(S)-4-(benzyl(4-benzylcarbamoyl-1-phenethylpiperidin-4-yl)amino)-3-benzyloxycarbonylamino-4-oxobutanoate (**53**): A solution of 1-phenethylpiperidin-4-one (0.22 mmol, 0.016 mL), benzylamine (0.44 mmol, 0.071 mL), Cbz-Asp(OMe)-OH (0.44 mmol, 122 mg) and benzyl isocyanide (0.44 mmol, 0.066 mL) in MeOH (2 mL) was reacted. After purification of the final residue, compound **53** (122 mg, 80%) was obtained as a white foam. ^1^H NMR (400 MHz, CDCl_3_) δ 7.20 (m, 20 H), 7.08 (d, *J* = 7.4 Hz, 1H), 6.71 (bt, *J* = 4.8 Hz, 1H), 5.02 (m. 1H), 4.9 (m, 3H), 4.76 (m, 3H), 4.32 (m, 2H), 3.47 (s, 3H), 2.55 (m, 8H), 1.97 (s, 2H), 2.37 (m, 2H). ^13^C NMR (101 MHz, CDCl_3_) δ 172.9, 172.5, 171.3, 171.2, 155.3, 138.7, 137.9, 135.9, 129.1, 128.7, 128.6, 128.5, 128.4, 128.3, 128.1, 127.7, 127.6, 127.2, 126.2, 126.1, 67.3, 64.9, 60.441, 59.9, 51.9, 50.3, 49.9, 49.6, 48.1, 43.6, 37.5. MS (ES+) *m/z*: 691.63 (M + 1)^+^. HPLC retention time = 8.40 min (99% analytical purity) (H_2_O/CH_3_CN from 15/85 to 0/100 in 5 min flow rate of 1 mL/min). Anal. calculated for C_41_H_46_N_4_O_6_ (C, H, N): C, 71.28; H, 6.71; N, 8.11. Found: C, 71.30; H, 6.55; N, 8.34.

Methyl(S)-4-((1-benzyl-4-benzylcarbamoylpiperidin-4-yl)(2-(phenylamino)ethyl)amino)-3-benzyloxycarbonylamino-4-oxobutanoate (**57**): 1-benzylpiperidin-4-one (0.22 mmol, 0.016 mL), *N^1^*-phenylethane-1,2-diamine (0.44 mmol, 0.071 mL), Cbz-Asp(OMe)-OH (0.44 mmol, 122 mg) and benzyl isocyanide (0.44 mmol, 0.066 mL) in MeOH (2 mL) reacted to give **57** (64 mg, 41%) as a white foam. ^1^H NMR (400 MHz, CDCl_3_) δ 7.32 (m, 18), 6.65 (t, *J* = 7.5 Hz, 1H), 6.52 (d, *J* = 7.5 Hz, 2H), 5.26 (m, *J* = 9.1 Hz, 1H), 4.97 (m, 4H), 4.33 – 4.11 (m, 3H), 3.76 (m, 2H), 3.46 (s, 3H), 3.40 (m, 4H), 2.52 (m, 9H). ^13^C NMR (101 MHz, CDCl_3_) δ 173.3, 172.8, 171.3, 155.6, 147.4, 138.7, 135.8, 129.7, 129.4, 128.8, 128.6, 128.5, 128.4, 128.4, 128.3, 128.1, 127.7, 127.6, 127.5, 127.2, 127.0, 118.2, 113.3, 67.4, 63.8, 62.4, 60.4, 52.0, 51.8, 50.2, 49.8, 48.9, 44.2, 43.8, 43.5, 37.4, 32.2. MS (ES+) *m/z* 706.72 (M + 1)^+^. HPLC retention time = 8.61 min (95% analytical purity) (H_2_O/CH_3_CN from 15/85 to 0/100 in 5 min flow rate of 1 mL/min). Anal. calculated for C_41_H_47_N_5_O_6_ (C, H, N): C, 69.77; H, 6.71; N, 9.92. Found: C, 69.81; H, 6.54; N, 9.73.

Methyl(S)-4-((4-benzylcarbamoyl-1-phenethylpiperidin-4-yl)(2-(phenylamino)ethyl)amino)-3-benzyloxycarbonylamino-4-oxobutanoate (**58**): 1-phenethylpiperidin-4-one (0.22 mmol, 0.016 mL), *N^1^*-phenylethane-1,2-diamine (0.44 mmol, 0.071 mL), Cbz-Asp(OMe)-OH (0.44 mmol, 122 mg) and benzyl isocyanide (0.44 mmol, 0.066 mL) in MeOH (2 mL) reacted according to the general procedure to give, after purification, a white foam that was identified as compound **58** (32 mg, 20%). ^1^H NMR (400 MHz, CDCl_3_) δ 7.13 (m, 18H), 6.66 (t, *J* = 7.5 Hz, 1H), 6.52 (d, *J* = 7.5 Hz, 2H), 5.29 (m, 1H), 5.23 (s, 1H), 5.02 (d, *J* = 12.0 Hz, 1H), 4.93 (d, *J* = 12.0 Hz, 1H), 4.92 (m, 1H), 4.14 (m, 3H), 3.77 (m, 2H), 3.47 (s, 3H), 3.40 (m, 2H), 2.73 (m, 9H), 2.49 (m, 4H). ^13^C NMR (101 MHz, CDCl_3_) δ 206.9, 173.2, 172.9, 171.3, 155.6, 147.4, 139.9, 138.7, 135.8, 129.4, 128.7, 128.6, 128.5, 128.5, 128.4, 128.1, 127.8, 127.2, 126.2, 118.3, 113.3, 77.4, 77.2, 77.0, 76.7, 67.4, 63.9, 59.9, 52.0, 50.2, 49.9, 48.9, 44.2, 43.8, 43.5, 37.7, 33.7, 32.7, 30.9. MS (ES+) *m/z*: 720.80 (M + 1)^+^. HPLC retention time = 8.68 min (97% analytical purity) (H_2_O/CH_3_CN from 15/85 to 0/100 in 5 min flow rate of 1 mL/min). Anal. calculated for C_42_H_49_N_5_O_6_ (C, H, N): C, 70.08; H, 6.86; N, 9.73. Found: C, 70.29; H, 6.51; N, 9.93.

Methyl(S)-4-((4-benzylcarbamoyl-1-phenethylpiperidin-4-yl)(4-methylbenzyl)amino)-3-benzyloxycarbonylamino-4-oxobutanoate (**59**): Reaction of 1-phenethylpiperidin-4-one (0.22 mmol, 0.016 mL), 4-methylbenzylamine (0.44 mmol, 0.071 mL), Cbz-Asp(OMe)-OH (0.44 mmol, 122 mg) and benzyl isocyanide (0.44 mmol, 0.066 mL) in MeOH (2 mL) gave **59** (112 mg, 72%) as a white foam. ^1^H RMN (400 MHz, DMSO-d6) δ 7.22 (m, 19H), 4.99 (dd, *J* = 16.0, 12.0 Hz, 2H,), 4.83 (d, *J* = 16.0 Hz, 1H), 4.80 (t, *J* = 8.1 Hz, 1H), 4.71 (d, *J* = 16.0 Hz, 1H), 4.28 (d, *J* = 8.1 Hz, 2H,), 3.58 (s, 3H), 3,09 (s, 2H), 2.85 (dd, 2H), 2.53 (m, 4H), 2.30 (s, 3H), 1.87 (2H, td, *J* = 12.2, 4.4 Hz, 4H), 1.72 (td, 2H, *J* = 12.2, 4.4 Hz, 4H). ^13^C RMN (100 MHz, CDCl_3_) δ 172.8, 172.5, 171.2, 155.3, 140.2, 138.7, 137.2, 136.0, 134.8, 129.7, 128.7, 128.6, 128.5, 128.4, 128.3, 128.1, 127.7, 127.2, 126.1, 126.0, 67.2, 65.0, 60.0, 51.9, 50.3, 49.9, 49.6, 47.8, 43.6, 37.5, 33.6, 32.8, 32.1, 21.1. MS (ES+) *m/z*: 705.72 (M + 1)^+^. HPLC retention time = 8.76 min (99% analytical purity) (H_2_O/CH_3_CN from 15/85 to 0/100 in 5 min flowrate of 1 mL/min). Anal. calculated for C_42_H_48_N_4_O_6_ (C, H, N): C, 71.57; H, 6.86; N, 7.95. Found: C, 71.42; H, 6.99; N, 7.74.

#### 4.1.3. General Ugi Reaction Protocol followed by Alkylation (Method B)

To a solution of piperidin-4-one (0.18 mmol) in methanol (2 mL) were added 2 equivalents of benzylamine, 2 equivalents of benzyl isocyanide and the corresponding amino acid (2 equivalents). After stirring the reaction for four days at room temperature, HCl in MeOH (1.2 M) was added and stirred at room temperature for 30 additional minutes. The solvent was removed, the residue redissolved in ethyl acetate and then successively washed with saturated NaHCO_3_ (3 × 10 mL) and brine (3 × 10 mL). The organic phase was dried (MgSO_4_), filtered and then evaporated to dryness. The Ugi adduct, thus obtained, was used in the next step without further purification. It was dissolved in DMF (5 mL) and alkylated by reaction with 1.2 equivalents of the corresponding alkyl bromide in the presence of 1.5 equivalents of K_2_CO_3_. The reaction mixture was stirred at 50 °C for 3 h. It was then neutralized with AcOH (0.3 mL), washed with brine, and dried over anhydrous MgSO_4_ and filtered. The final crude was purified by flash column chromatography using a mixture of Hexane/EtOAc (1:1 to 0:1) to give the novel *N*-benzyl 4,4-disubstituted piperidine analogues **54**–**56** and **60**–**63.**

Methyl(S)-4-(benzyl(4-benzylcarbamoyl-1-(4-methylphenethyl)piperidin-4-yl)amino)-3-benzyloxycarbonylamino-4-oxobutanoate (**54**): Following Method B, 4-piperidone monohydrate hydrochloride (0.18 mmol, 28 mg), benzylamine (0.36 mmol, 0.058 mL), Cbz-Asp(OMe)-OH (0.36 mmol, 101 mg) and benzyl isocyanide (0.36 mmol, 0.054 mL) in MeOH (2 mL) were reacted. Next, after treating the Ugi adduct with 4-methylphenethyl bromide (0.22 mmol, 44 mg) and with K_2_CO_3_ (0.27 mmol, 37 mg) in DMF followed by purification, **54** (30 mg, 24%) was obtained as a white foam. ^1^H NMR (400 MHz, CDCl_3_) δ 7.25 (m, 11H), 7.15 (m, 4H), 7.02 (d, *J* = 7.8 Hz, 2H), 6.97 (d, *J* = 7.8 Hz, 2H), 6.73 (s, 1H), 4.99 (d, *J* = 12.1 Hz, 1H), 4.89 (d, *J* = 12.1 Hz, 1H), 4.83 (m, 1H), 4.74 (d, *J* = 18.0 Hz, 1H), 4.30 (dd, *J* = 15.1, 5.8 Hz, 1H), 4.17 (m, 2H), 3.77 (m, 1H), 3.45 (s, 3H), 3.22 (m, 3H), 2.91 (m, 4H), 2.59 (dd, *J* = 16.4, 4.8 Hz, 1H), 2.42 (s, 1H), 2.25 (d, *J* = 7.3 Hz, 1H), 2.22 (s, 3H), 1.99 (s, 4H). ^13^C NMR (101 MHz, CDCl_3_) δ 178.1, 175.9, 172.9, 171.6, 155.4, 138.4, 137.7, 136.6, 135.7, 129.4, 129.3, 128.8, 128.6, 128.5, 128.4, 128.2, 127.8, 127.3, 127.2, 126.5, 67.5, 58.3, 52.0, 49.4, 47.9, 43.5, 37.1, 36.4, 30.4, 29.7, 21.0, 18.5. MS (ES+) *m/z*: 705.57 (M+1)^+^. HPLC retention time = 8.70 min (99% analytical purity) (H_2_O/CH_3_CN from 15/85 to 0/100 in 5 min flow rate of 1 mL/min). Anal. calculated for C_42_H_48_N_4_O_6_ (C, H, N): C, 71.57; H, 6.86; N, 7.95; O, 13.62. Found: C, 71.84; H, 6.91; N, 7.70; O, 13.49.

Methyl(S)-4-(benzyl(4-benzylcarbamoyl-1-(4-nitrophenethyl)piperidin-4-yl)amino)-3-benzyloxycarbonylamino-4-oxobutanoate (**55**): According to general method B, 4-piperidone monohydrate hydrochloride (0.18 mmol, 28 mg), benzylamine (0.36 mmol, 0.058 mL), Cbz-Asp(OMe)-OH (0.36 mmol, 101 mg) and benzyl isocyanide (0.36 mmol, 0.054 mL) in MeOH (2 mL) were reacted. To the Ugi adduct, intermediate 4-nitrophenethyl bromide (0.22 mmol, 51 mg) and K_2_CO_3_ (0.27 mmol, 37 mg) in DMF were added. Purification of the final residue yielded compound **55** (34 mg, 26%) as a white foam. ^1^H NMR (500 MHz, CDCl_3_) δ 8.13 (d, *J* = 8.4 Hz, 2H), 7.35 (m, 15H), 7.24 (d, *J* = 7.8 Hz, 3H), 6.79 (s, 1H), 5.33 (s, 1H), 5.05 (d, *J* = 12.2 Hz, 1H), 4.98 (d, *J* = 12.2 Hz, 1H), 4.87 (m, 1H), 4.81 (d, *J* = 17.7 Hz, 1H), 4.38 (m, 2H), 3.54 (s, 3H), 2.82 (s, 4H), 2.59 (dd, *J* = 47.7, 13.6 Hz, 4H), 1.73 (s, 4H), 1.26 (s, 2H). 1^3^C NMR (126 MHz, CDCl_3_) δ 172.8, 171.4, 155.3, 138.6, 137.8, 135.8, 129.5, 129.2, 128.7, 128.6, 128.5, 128.4, 128.2, 127.7, 127.5, 127.3, 126.3, 123.8, 77.3, 77.3, 77.2, 77.0, 76.9, 76.8, 67.4, 52.0, 50.3, 49.7, 49.4, 48.1, 43.5, 37.3, 29.7. MS (ES+) *m/z*: 736.73 (M + 1)^+^. HPLC retention time = 8.42 min (99% analytical purity) (H_2_O/CH_3_CN from 15/85 to 0/100 in 5 min flow rate of 1 mL/min). Anal. calculated for C_41_H_45_N_5_O_8_ (C, H, N): C, 66.92; H, 6.16; N, 9.52. Found: C, 66.79; H, 6.22; N, 9.65.

Methyl(S)-4-(benzyl(4-benzylcarbamoyl-1-(4-fluorophenethyl)piperidin-4-yl)amino)-3-benzyloxycarbonylamino-4-oxobutanoate (**56**): Reactants: benzylamine (0.36 mmol, 0.058 mL), a solution of 4-piperidone monohydrate hydrochloride (0.18 mmol, 28 mg), Cbz-Asp(OMe)-OH (0.36 mmol, 101 mg) and benzyl isocyanide (0.36 mmol, 0.054 mL) in MeOH (2 mL). Subsequent treatment of adduct with 4-fluorophenethyl bromide (0.22 mmol, 45 mg) and with K_2_CO_3_ (0.27 mmol, 37 mg) in DMF afforded, after chromatography, a white foam that was compound **56** (28 mg, 22%). ^1^H NMR (400 MHz, CDCl_3_) δ 7.29 (m, 15H), 7.07 (dd, *J* = 8.7, 5.5 Hz, 2H), 6.92 (t, *J* = 8.7 Hz, 2H), 6.76 (t, *J* = 5.8 Hz, 1H), 5.37 (d, *J* = 8.9 Hz, 1H), 4.98 (q, *J* = 12.2 Hz, 2H), 4.80 (m, 3H), 4.40 (dd, *J* = 14.8, 6.0 Hz, 1H), 4.33 (dd, *J* = 14.8, 5.7 Hz, 1H), 3.52 (s, 3H), 2.75 (m, 6H), 2.61 (d, *J* = 5.2 Hz, 1H), 2.54 (m, 4H), 2.36 (m, 1H), 1.98 (m, 2H). ^13^C NMR (101 MHz, CDCl_3_) δ 172.9, 172.6, 171.4, 161.5 (d, *J_C-F_* = 243.3 Hz), 155.3, 138.7, 137.9, 135.9, 130.1 (d, J_C-F_ = 7.7 Hz), 129.1, 128.7, 128.6, 128.4, 128.2, 127.7, 127.7, 127.3, 126.3, 115.2 (d, *J_C-F_* = 20.9 Hz), 77.4, 77.3, 77.1, 76.8, 67.4, 59.9, 52.0, 50.3, 49.9, 49.6, 48.1, 43.6, 37.5. MS (ES+) *m/z*: 709.70 (M + 1)^+^. HPLC retention time = 8.47 min (97% analytical purity) (H_2_O/CH_3_CN from 15/85 to 0/100 in 5 min flowrate of 1 mL/min). Anal. calculated for C_41_H_45_FN_4_O_6_ (C, H, F, N): C, 69.47; H, 6.40; F, 2.68; N, 7.90. Found: C, 69.19; H, 6.55; F, 2.59; N, 7.73.

*Tert*-butyl(1-(benzyl(4-benzylcarbamoyl-1-(4-fluorobenzyl)piperidin-4-yl)amino)-1-oxo-3-phenylpropan-2-yl)carbamate (**60**): The reagents 4-piperidone monohydrate hydrochloride (0.18 mmol, 28 mg), benzylamine (0.36 mmol, 0.058 mL), Boc-Phe-OH (0.36 mmol, 96 mg) and benzyl isocyanide (0.36 mmol, 0.054 mL) in MeOH (2 mL) were reacted. 4-fluorobenzyl bromide (0.22 mmol, 42 mg) and K_2_CO_3_ (0.27 mmol, 37 mg) in DMF were then added to the obtained crude of reaction. Finally, **60** (57 mg, 47%) was obtained, by purification of the crude, as a white foam. ^1^H NMR (500 MHz, DMSO-d6) δ 7.83 (t, *J* = 5.9 Hz, 1H), 7.23 (m, 16H), 7.05 (m, 2H), 7.01 (d, *J* = 7.2 Hz, 2H), 4.89 (d, *J* = 18.5 Hz, 1H), 4.78 (d, *J* = 18.5 Hz, 1H), 4.32 (m, 1H), 3.27 (m, 2H), 4.26 (m, 2H), 2.91 (dd, *J* = 13.9, 4.0 Hz, 1H), 2.68 (dd, *J* = 13.9, 9.7 Hz, 1H), 2.55 (m, 1H), 2.40 (m, 4H), 2.24 (t, *J* = 11.6 Hz, 1H), 1.76 (td, *J* = 12.5, 4.0 Hz, 1H), 1.53 (m, 1H), 1.22 (s, 9H). ^13^C NMR (126 MHz, DMSO-d6) δ 173.8, 172.7, 161.6 (d, *J_C-F_* = 242.1 Hz), 155.8, 140.4, 139.4, 138.4, 134.9 (d, *J* = 3.2 Hz), 130.9 (d, *J_C-F_* = 7.9 Hz), 129.8, 129.7, 129.0, 128.5, 128.4, 127.6, 127.5, 127.4, 126.9, 126.9, 126.7, 124.9, 115.2 (d, *J_C-F_* = 21.0 Hz), 78.5, 78.4, 63.9, 61.2, 54.5, 49.9, 49.7, 48.1, 42.8, 37.8, 32.6, 32.2, 28.5. MS (ES+) *m/z*: 679.59 (M + 1)^+^. HPLC retention time = 9.13 min (96% analytical purity) (H_2_O/CH_3_CN from 15/85 to 0/100 in 5 min flowrate of 1 mL/min). Anal. calculated for C_41_H_47_FN_4_O_4_ (C, H, F, N): C, 72.54; H, 6.98; F, 2.80; N, 8.25. Found: C, 72.33; H, 6.71; F, 2.95; N, 8.01.

*Tert*-butyl(1-(benzyl(4-benzylcarbamoyl-1-(4-chlorobenzyl)piperidin-4-yl)amino)-1-oxo-3-phenylpropan-2-yl)carbamate (**61**): General procedure B was followed with a solution of Boc-Phe-OH (0.36 mmol, 96 mg), benzylamine (0.36 mmol, 0.058 mL), benzyl isocyanide (0.36 mmol, 0.054 mL) and 4-piperidone monohydrate hydrochloride (0.18 mmol, 28 mg), in MeOH (2 mL). The Ugi intermediate was treated with 4-chlorobenzyl bromide (0.22 mmol, 41 mg) and with K_2_CO_3_ (0.27 mmol, 37 mg) in DMF. The final residue was purified to give a white foam that resulted in compound **61** (68 mg, 54%). ^1^H NMR (500 MHz, DMSO-d6) δ 7.84 (t, *J* = 5.9 Hz, 1H), 7.34 – 7.23 (m, 11H), 7.18 (m, 7H), 7.01 (d, *J* = 7.2 Hz, 2H), 4.90 (d, *J* = 18.5 Hz, 1H), 4.78 (d, *J* = 18.5 Hz, 1H), 4.31(m, 1H), 4.22 (m, 2H), 3.28 (s, 2H), 2.92 (dd, *J* = 13.8, 4.0 Hz, 1H), 2.69 (dd, *J* = 13.8, 9.7 Hz, 1H), 2.56 (m, 1H), 2.40 (m, 4H), 2.25 (t, *J* = 11.6 Hz, 1H), 1.77 (m, 1H), 1.53 (m, 1H), 1.22 (s, 9H). ^13^C NMR (126 MHz, DMSO-d6) δ 173.8, 172.7, 155.8, 140.4, 139.4, 139.1, 138.4, 137.9, 131.8, 130.9, 129.8, 129.7, 129.0, 128.5, 128.5, 128.4, 127.6, 127.5, 127.4, 126.9, 126.9, 126.7, 124.9, 78.5, 78.4, 63.9, 61.2, 55.4, 54.5, 49.9, 49.7, 48.1, 42.8, 37.8, 32.6, 32.2, 28.5. MS (ES+) *m/z*: 695.46 (M)^+^. HPLC 9.34 min (98%) (H_2_O/CH_3_CN from 15/85 to 0/100 in 5 min flowrate of 1 mL/min). Anal. for C_41_H_47_ClN_4_O_4_ (C, H, Cl, N C, 70.83; H, 6.81; Cl, 5.10; N, 8.06. Found: C, 70.99; H, 6.65; Cl, 5.39; N, 8.34.

*Tert*-butyl(1-(benzyl(4-benzylcarbamoyl-1-(4-methoxybenzyl)piperidin-4-yl)amino)-1-oxo-3-phenylpropan-2-yl)carbamate (**62**): Method B was used with 4-piperidone monohydrate hydrochloride (0.18 mmol, 28 mg), benzylamine (0.36 mmol, 0.058 mL), Boc-Phe-OH (0.36 mmol, 96 mg) and benzyl isocyanide (0.36 mmol, 0.054 mL) in MeOH (2 mL). Next, the Ugi adduct intermediate was treated with 4-methoxybenzyl bromide (0.22 mmol, 44 mg) and with K_2_CO_3_ (0.27 mmol, 37 mg) in DMF. The residue was chromatographed to afford **62** (46 mg, 37%) as a white foam. ^1^H NMR (500 MHz, DMSO-d6) δ 7.82 (t, *J* = 5.9 Hz, 1H), 7.35 – 7.24 (m, 9H), 7.22 – 7.11 (m, 5H), 7.03 (m, 4H), 6.80 (d, *J* = 8.6 Hz, 2H), 4.89 (d, *J* = 18.5 Hz, 1H), 4.77 (d, *J* = 18.5 Hz, 1H), 4.30 (m, 1H), 4.25 (m, 2H), 3.69 (s, 3H), 3.21 (s, 2H), 2.91 (dd, *J* = 13.9, 4.0 Hz, 1H), 2.68 (dd, *J* = 13.9, 9.6 Hz, 1H), 2.54 (m, 1H), 2.38 (m, 4H), 2.22 (m, 1H), 1.73 (m, 1H), 1.51 (m, 1H), 1.22 (s, 9H). ^13^C NMR (126 MHz, DMSO-d6) δ 173.7, 172.8, 158.6, 155.8, 140.4, 139.5, 138.4, 130.6, 130.4, 129.7, 129.0, 128.5, 128.4, 127.5, 127.4, 126.9, 126.9, 126.7, 113.9, 78.5, 78.4, 63.9, 61.6, 55.4, 54.5, 49.9, 49.7, 48.1, 42.8, 37.9, 32.6, 32.2, 28.5. MS (ES+) *m/z*: 691.47 (M)^+^. HPLC retention time = 9.15 min (96% analytical purity) (H_2_O/CH_3_CN from 15/85 to 0/100 in 5 min flow rate of 1 mL/min). Anal. calculated for C_42_H_50_N_4_O_5_ (C, H, N): C, 73.02; H, 7.29; N, 8.11. Found: C, 73.21; H, 7.52; N, 8.03

*Tert*-butyl(1-(benzyl(4-benzylcarbamoyl-1-(4-nitrobenzyl)piperidin-4-yl)amino)-1-oxo-3-phenylpropan-2-yl)carbamate (**63**): Benzylamine (0.36 mmol, 0.058 mL), Boc-Phe-OH (0.36 mmol, 96 mg), benzyl isocyanide (0.36 mmol, 0.054 mL) and 4-piperidone monohydrate hydrochloride (0.18 mmol, 28 mg) were reacted in MeOH (2 mL) and then treated with 4-nitrobenzyl bromide (0.22 mmol, 47 mg) and with K_2_CO_3_ (0.27 mmol, 37 mg) in DMF. Finally, after work-up and purification, compound **63** (71 mg, 56%) was obtained as a white foam. ^1^H NMR (500 MHz, DMSO-d6) δ 8.11 (d, *J* = 8.5 Hz, 2H), 7.85 (d, *J* = 5.9 Hz, 1H), 7.43 (d, *J* = 8.5 Hz, 2H), 7.35 – 7.24 (m, 9H), 7.22 – 7.11 (m, 5H), 7.02 (d, *J* = 7.2 Hz, 2H), 4.91 (d, *J* = 18.4 Hz, 1H), 4.80 (d, *J* = 18.4 Hz, 1H), 4.34 (m, 1H), 4.27 (t, *J* = 6.3 Hz, 2H), 3.43 (s, 2H), 2.92 (dd, *J* = 13.9, 4.0 Hz, 1H), 2.69 (dd, *J* = 13.9, 9.7 Hz, 1H), 2.53 (m, 1H), 2.41 (m, 4H), 2.30 (m, 1H), 1.85 (m, 1H), 1.56 (m, 1H), 1.22 (s, 9H). ^13^C NMR (126 MHz, DMSO-d6) δ 173.8, 172.7, 155.8, 147.3, 146.9, 140.4, 139.4, 138.4, 130.1, 129.7, 129.0, 128.5, 128.4, 127.5, 127.4, 126.9, 126.9, 126.7, 123.7, 78.5, 78.4, 63.8, 61.2, 54.5, 50.1, 49.84, 42.8, 37.8, 32.7, 32.2, 28.5. MS (ES+) *m/z*: 706.41 (M + 1)^+^. HPLC retention time = 9.06 min (95% analytical purity) (H_2_O/CH_3_CN from 15/85 to 0/100 in 5 min flow rate of 1 mL/min). Anal. calculated for C_41_H_47_N_5_O_6_ (C, H, N): C, 69.77; H, 6.71; N, 9.92. Found: C, 69.91; H, 6.49; N, 9.81.

### 4.2. Biological Procedures

#### 4.2.1. Cytopathic Effect Reduction Assay with HCoV-229E

We purchased HCoV-229E and human embryonic lung (HEL) fibroblast cells from ATCC (VR-740 and CCL-137). To conduct the cytopathic effect (CPE) reduction assay [57], confluent HEL cell cultures in 96-well plates were exposed to serial compound dilutions and immediately infected with HCoV-229E (MOI: 100 CCID_50_ per well). At the same time, we added the compounds to mock-infected plates to measure cytotoxicity. After five days of incubation at 35 °C, the plates were submitted to microscopic scoring of CPE (virus-infected plates) and compound cytotoxicity (mock-infected plates). To quantify the results by MTS cell viability assay, the medium was replaced by CellTiter 96^®^ AQ_ueous_ MTS Reagent (from Promega) and, on the next day, optical density at 490 nm was measured in a plate reader. The 50% effective concentration (EC_50_), based on microscopy or MTS assay, and the 50% cytotoxic concentration (CC_50_) by MTS assay were calculated using reported formulas [58]. The cytotoxicity estimated by microscopy was expressed as the minimal cytotoxic concentration (MCC) causing visible alterations in cell morphology. We included two reference compounds: GS-441524 (the nucleoside form of remdesivir; from Carbosynth) and K22 [(*Z*)-*N*-[3-[4-(4-bromophenyl)-4-hydroxypiperidin-1-yl]-3-oxo-1-phenylprop-1-en-2-yl]benzamide [36]; from ChemDiv].

#### 4.2.2. Time-Of-Compound Addition Assay with HCoV-229E

The compounds were added to confluent HEL cells in a 96-well plate at different time points (= -0.5, 0.5, 2, 4, 6 or 8 h) p.i. with HCoV-229E (MOI: 100) [57]. Besides GS-441524 and K22, we included three extra reference compounds: bafilomycin A_1_ (from Cayman), E64d (from Sigma-Aldrich) and GC376 (from Carbosynth). At 16 h p.i., we removed the supernatant and washed the cells twice with ice-cold PBS. Next, the cells were lysed with 22 µL of a 1:10 mixture of lysis enhancer and resuspension buffer (CellsDirect One-Step RT-qPCR kit; Invitrogen). After 10 min on ice, the lysates were collected and heated for 10 min at 75 °C. After exposing the samples to DNase, the viral RNA copy number was determined by one-step RT-qPCR. A total of 5 µL of lysate was mixed with 10 µL of RT-qPCR mix containing Reaction mix with ROX, MgSO_4_ and Superscript III RT/Platinum Taq mix (all from a CellsDirect One-Step RT-qPCR kit), as well as a probe and primer pair targeting the HCoV-229E N-gene [58]. The RT-qPCR program consisted of: 15 min at 50 °C; 2 min at 95 °C; and 40 cycles of 15 s at 95 °C and 45 s at 60 °C. To enable absolute quantification, we included an N-gene plasmid standard. The data were expressed relative to the RNA copy number measured in the virus control that received no compound.

#### 4.2.3. Antiviral Evaluation against SARS-CoV-2

We used a SARS-CoV-2 strain of clade 20A.EU2 / B.1.160 [GISAID accession number EPI_ISL_888706 and generous gift from P. Maes (Leuven)] that was isolated in early 2020 and bears spike variation G614. The A549 cells expressing ACE and TMPRSS2 (designated as A549-AT cells in the text) were purchased from Invivogen and cultured under hygromycin and puromycin. For the antiviral assay, we seeded the cells in 96-well plates at 15,000 cells per well. One day later, we added serial dilutions of the compounds together with the virus (MOI: 100 CCID_50_ per well). Two hours later, the supernatants were discarded to remove non-infectious virus particles and fresh compound was added. To estimate compound cytotoxicity, a mock-infected plate was included in parallel. After three days of incubation at 37 °C, this mock plate underwent MTS cell viability analysis, similar to the method described above, yielding the CC_50_ values. At the same time, we collected the supernatant samples to measure the viral genome copy number by qRT-PCR using the CellsDirect One-Step RT-qPCR kit (Invitrogen). The SARS-CoV-2 N-gene directed primers and probe were purchased from IDT (US CDC 2019-nCoV_N1) along with the plasmid standard (2019-nCoV_N positive control plasmid). All technical details can be found elsewhere [39]. The results were expressed as fold reductions in viral load (compound-treated compared to untreated virus control) and used to construct dose–response curves (nonlinear least-squares regression analysis, Graphpad Prism software). The EC_90_ and EC_99_ values, i.e., compound concentration producing 10- and 100-fold reductions in viral load, were determined by interpolation on the dose–response curves (Graphpad Prism).

#### 4.2.4. Production of SARS-CoV-2 Recombinant Proteins

The nsp12, nsp7 and nsp8 components of the SARS-CoV-2 RdRp were purified separately and the complex was re-assembled as described in [35], with modification as described in [26]. The SARS-CoV-2 nsp14, nsp10 and nsp16 coding sequences were cloned in fusion with an N-terminal hexa-histidine tag in pET28 plasmids. The proteins were expressed in *E. coli* and purified by two-step chromatography. Briefly, C2566 cells expressing the recombinant proteins were lysed by sonication in Tris buffer [i.e., 50 mM Tris pH 6.8; 300 mM NaCl; 5 mM MgCl_2_; and 1 mM β-mercaptoethanol] supplemented with 10 mM imidazole, 0.25 mg/mL Lysozyme, 10 μg/mL DNase and 1 mM PMSF. To purify the protein through affinity chromatography, the resin (IMAC Cobalt resin 480; Thermo Scientific, Waltham, MA, USA) was washed with an increased concentration of salt (1 M NaCl) and imidazole (20 mM), then eluted in Tris buffer with 250 mM of imidazole. Finally, the protein was purified by size-exclusion with a GE Superdex S200 using the same Tris buffer as above.

6xHis-tagged SARS-CoV-2 nsp5 protein was produced in *E. coli* as previously described [31]. After transforming the pGEX-6P-1 plasmid in *E. coli* BL21 DE3 gold, nsp5 production was stimulated by overnight induction with 250 μM IPTG at a temperature of 17 °C. After centrifugation, the bacteria were resuspended in buffer consisting of: 50 mM Tris pH 8; 300 mM NaCl; 5 mM MgSO_4_; 1 mM PMSF; 10 μg/mL DNAse I; 0.25 mg/mL Lysozyme; 0.1%Triton X-100; and 10% glycerol. The cells were lysed by three cycles of sonication and centrifugation. The nsp5 protein was bound to Co-NTA agarose beads and eluted in lysis buffer containing 250 mM of imidazole. Next, the protein product was concentrated on a Vivaspin 20 centrifugal concentrator (10 kDa cut-off; GE Healthcare) and imidazole was removed by dialysis. Finally, the purified nsp5 was stored at −80 °C with 50% glycerol.

#### 4.2.5. Enzymatic Assays

**MTase assays.** All details can be found elsewhere [33,45]. After mixing the enzymes with the test compound dissolved in DMSO (5% final DMSO), RNA substrate and S-adenosylmethionine were added and the samples were incubated at 30 °C. To stop the reaction after 30 min, the mixtures were diluted 10-fold in ice-cold water. Next, they were transferred to diethylaminoethyl (DEAE) filter mats (Perkin Elmer) using a Filtermat Harvester (Packard Instruments). After extensive washing [i.e., twice with 10 mM ammonium formate pH 8.0, twice with water and once with ethanol] and soaking in scintillation fluid, the mats were submitted to ^3^H-counting in a MicroBeta TriLux apparatus (Perkin Elmer). To calculate the IC_50_ value [=compound concentration causing 50% reduction in enzyme activity], the readout values were normalized and fitted with GraphPad Prism software using the equation Y = 100/(1 + (X/IC_50_)^HillSlope).

**M^pro^ enzymatic assay.** The fluorescence resonance energy transfer (FRET)-based protease assay was performed in black 384-well plates. Briefly, serial dilutions of the compounds were incubated with 80 nM nsp5 protein and 5 μM peptide substrate (Dabcyl-KTSAVLQ↓SGFRKM-Edans-NH_2_; Genscript) [47] in buffer consisting of: 20 mM HEPES pH 6.5; 120 mM NaCl; 0.4 mM EDTA; 4 mM dithiothreitol; and 10% glycerol. The final DMSO content was 0.5%. Cleavage of the substrate was monitored for 40 min by measuring the increasing fluorescence in a Tecan Safire 2 plate reader (wavelength settings: excitation at 335 nm and emission at 493 nm). For each condition, enzyme activity was derived from the slope for the linear part of the curve and expressed relative to the activity in the condition receiving no compound. To determine the IC_50_ values, GraphPad Prism software was used to plot the % of enzyme activity against compound concentration and then fit the curves according to the equation Y = 100/(1 + (X/IC_50_)^HillSlope^).

### 4.3. Computational Methods

#### 4.3.1. Protein Models

Entries 6XHM [59], 7BQY [60] and 7TLL [53], reporting the X-ray crystal structures of SARS-CoV-2 M^pro^ as found in the covalent complexes formed with ligands PF-00835231, N3, and nirmatrelvir (PF-07321332), respectively, were retrieved from the Protein Data Bank in Europe – Knowledge Base (PDBe-KB) [61,62]. The protein backbone of these three structures was very similar overall, with the exception of the region encompassing ^187^Asp-Ala^193^ which adapts in response to the different ligands, hence the need to consider multiple conformations for ligand docking experiments. To study the dynamics of the enzyme dimer, we also modeled PDB entry 5R8T reporting the SARS-CoV-2 M^pro^ dimer structure solved at 1.27 Å resolution and employed for fragment screening [63]. In all cases, crystallographic water molecules were kept and histidine residues were considered to be protonated on N^ε^, except for His164 (protonated on N^δ^) and His80 (doubly protonated).

#### 4.3.2. Ligand Preparation

Entry QUWNIH from the Cambridge Structural Database [64] reporting the X-ray crystal structure of a macrocycle containing a 1,4,4-trisubstituted piperidine scaffold [65] provided the template for ligand construction making use of the editing facilities implemented in the molecular graphics program PyMOL (Schrodinger, L. L. C., The PyMOL Molecular Graphics System, v. 1.8, 2015). The geometries of ligands **34**, **45** and **52**, taken as representatives of the whole series, were optimized by means of the AM1-BCC Hamiltonian available in the *sqm* program [66] and the *peptide_corr = 1* keyword. This procedure also procured atom point charges that were used in conjunction with generalized AMBER force field (*gaff*) parameters for small molecules [67].

#### 4.3.3. Automated Ligand Docking

A three-dimensional cubic grid consisting of 65 × 65 × 65 points with a spacing of 0.375 Å centered at the catalytic Cys145 of one subunit was defined for ligand docking. AutoDock Vina 1.2.0 [68] was used to generate up to 10 feasible binding poses for each ligand studied allowing flexibility for the side chains of residues Leu27, His41, Met49, Asn142, Cys145, His163, Met165, Glu166 and Gln189. The best poses were ranked on the basis of results from intra- and intermolecular energy evaluations. As a control of docking performance, we used PF-00835231, N3 and nirmatrelvir (PF-07321332)—in their reactive, prebonded forms—and protein structures 6XHM and 7BQY after replacing Cys145 with Ala145. By following this simple procedure, we could assess that the near-attack conformations found by AutoDock Vina were very similar to the covalently bonded poses found in the respective crystals, provided the ^187^Asp-Ala^193^ segment was properly positioned beforehand.

#### 4.3.4. Energy Minimization and Molecular Dynamics Simulations

SARS-CoV-2 M^pro^ was simulated as a dimer for informative purposes for a duration of 150 ns, but for computational efficiency each of the selected complexes comprised just one enzyme monomer and the corresponding ligand. Each binary complex was immersed in a box of TIP3P water molecules that extended 12 Å away from any protein or ligand atom and neutralized by addition of three K^+^ ions. The *leaprcff14SB* AMBER force field [69] and the graphics processing unit (GPU)-based implementation of the *pmemd.cuda* module of AMBER18 in the single-precision−fixed-precision (SPFP) mode were used. A cutoff distance of 9 Å was defined for the nonbonded interactions. The SHAKE algorithm was applied to all hydrogen-involving bonds and the integration time step was 2 fs. A weak harmonic restraint of 0.5 kcal·mol^−1^·Å^−2^ was initially imposed on the protein’s Cα atoms (except for those in the ^187^Asp-Ala^193^ stretch) to promote ligand, water and counterion equilibration. Trajectory snapshots were saved every 0.1 ns for further analysis. In addition, coordinates saved every 5 ns were cooled down from 303 to 273 K in the absence of any restraints over a 1-ns period and then energy-minimized until the root-mean-square of the Cartesian elements of the gradient was less than 0.01 kcal mol^−1^ Å^−1^. The degree of convergence of these “frozen” complexes was used to assess the goodness of the binding mode [70].

## Figures and Tables

**Table 1:**
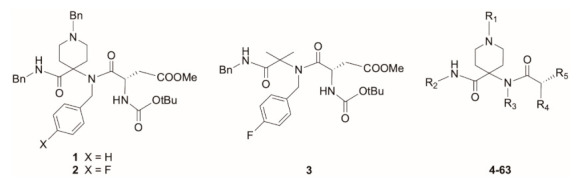
Anti-HCoV-229E activity of 1,4,4-trisubstituted piperidine analogues of compounds **1** and **2**.

Compound	R_1_	R_2_	R_3_	R_4_	R_5_	Antiviral EC_50_ (µM) ^a^		Cytotoxicity (µM) ^b^		Selectivity Index ^c^	
						Microscopy	MTS	MCC	CC_50_	Microscopy	MTS
**1** ^d^	Bn	Bn	Bn	NHBoc	CH_2_COOMe	>100	>100	>100	>100		
**2** ^d^	Bn	Bn	4-F-Bn	NHBoc	CH_2_COOMe	7.8 ± 2.7	7.4 ± 2.5	100	44 ± 8	13	6
**Subseries 1: modifications at R_1_**											
**3** ^e^	-	-	-	-	-	>100	>100	>100	>100		
**4** ^e^	H	Bn	4-F-Bn	NHBoc	CH_2_COOMe	50 ± 18	78 ± 14	100	66 ± 7		
**5** ^e^	Me	Bn	4-F-Bn	NHBoc	CH_2_COOMe	22 ± 9	22 ± 7	>100	92 ± 4		
**6** ^e^	Chx	Bn	4-F-Bn	NHBoc	CH_2_COOMe	22 ± 9	14 ± 1	100	85 ± 9		
**7** ^e^	Ph	Bn	4-F-Bn	NHBoc	CH_2_COOMe	43 ± 3	>100	>100	>100		
**8** ^e^	Bn(Me) piperidinium salt	Bn	4-F-Bn	NHBoc	CH_2_COOMe	>100	>100	>100	>100		
**9** ^e^	3-F-Bn	Bn	4-F-Bn	NHBoc	CH_2_COOMe	>100	>100	>100	>100		
**10** ^e^	3,5-diF-Bn	Bn	4-F-Bn	NHBoc	CH_2_COOMe	15 ± 1	>100	70	10 ± 2		
**11** ^e^	(CH_2_)_2_Ph	Bn	4-F-Bn	NHBoc	CH_2_COOMe	3.2 ± 0.1	4.4 ± 0.8	100	38 ± 2	31	
**Subseries 2: modifications at R_2_**											
**12** ^e^	Bn	^t^Bu	4-F-Bn	NHBoc	CH_2_COOMe	11	11	≥100	77		
**13** ^e^	Bn	Chx	4-F-Bn	NHBoc	CH_2_COOMe	>100	>100	>100	>100		
**14** ^e^	Bn	CH_2_SO_2_Ph-4-Me	4-F-Bn	NHBoc	CH_2_COOMe	57 ± 18	30 ± 6	100	78 ± 11		
**15**	Bn	^t^Bu	Bn	NHBoc	CH_2_COOMe	25 ± 0	25 ± 0	>100	78 ± 7		
**16**	Bn	Chx	Bn	NHBoc	CH_2_COOMe	16 ± 3	10 ± 0	≥40	42 ± 4		
**17** ^d^	Bn	CH_2_COOMe	Bn	NHBoc	CH_2_COOMe	68 ± 3	75 ± 8	>100	>100		
**Subseries 3: modifications at R_3_ and/or R_4_**											
**18** ^d^	Bn	Bn	H	NHBoc	CH_2_COOMe	55 ± 16	>100	≥100	48 ± 8		
**19** ^d^	Bn	Bn	Me	NHBoc	CH_2_COOMe	14 ± 1	>100	100	18 ± 2		
**20** ^d^	Bn	Bn	CH(Me)(Et)	NHBoc	CH_2_COOMe	14 ± 1	13 ± 1	100	31 ± 3		
**21** ^d^	Bn	Bn	Cyclopropyl	NHBoc	CH_2_COOMe	12 ± 0.4	> 100	50	14 ± 3		
**22** ^e^	Bn	Bn	4-Me-Bn	NHBoc	CH_2_COOMe	3.1 ± 0.0	4.3 ± 0.6	75	31 ± 10	24	
**23** ^e^	Bn	Bn	4-NO_2_-Bn	NHBoc	CH_2_COOMe	3.1 ± 0.0	4.0 ± 0.6	75	49 ± 6	24	12
**24** ^e^	Bn	Bn	4-CF_3_-Bn	NHBoc	CH_2_COOMe	3.1 ± 0.0	4.2 ± 1.0	25	11 ± 1		
**25** ^e^	Bn	Bn	4-Cl-Bn	NHBoc	CH_2_COOMe	3.1 ± 0.0	3.2 ± 0.2	50	12 ± 0		
**26** ^e^	Bn	Bn	2-F-Bn	NHBoc	CH_2_COOMe	12 ± 1	15 ± 2	100	44 ± 1		
**27** ^e^	Bn	Bn	3-F-Bn	NHBoc	CH_2_COOMe	10 ± 3	14 ± 1	100	46 ± 4	10	
**28** ^e^	Bn	Bn	3,4-F-Bn	NHBoc	CH_2_COOMe	6.0± 2.6	3.7 ± 1.0	44	23 ± 11		
**29** ^d^	Bn	Bn	PhNH(CH_2_)_2_	NHBoc	CH_2_COOMe	3.0 ± 0.1	3.2 ± 0.4	75	36 ± 9	25	11
**30** ^e^	Bn	Bn	4-F-Bn	H	CH_2_COOMe	13 + 0	13 ± 2	100	64 ± 3		
**31** ^e^	Bn	Bn	4-F-Bn	NH_2_	CH_2_COOMe	3.3 ± 0.2	2.8 ± 0.5	11	4.9 ± 1.5		
**32** ^e^	Bn	Bn	Bn	NH_2_	CH_2_COOMe	55 ± 3	62 ± 7	>100	>100		
**33** ^d^	Bn	Bn	Bn	NHCbz	CH_2_COOMe	3.0 ± 0.1	3.3 ± 0.4	>100	>100	>33	>30
**34** ^e^	Bn	Bn	4-F-Bn	NHCbz	CH_2_COOMe	3.1 ± 0.1	3.4 ± 0.4	81	75 ± 18	26	22
**35** ^d^	Bn	Bn	Bn	NHFmoc	CH_2_COOMe	36 ± 14	26 ± 9	>100	>100		
**36** ^d^	Bn	Bn	CH(Me)(Et)	NHCbz	CH_2_COOMe	9.2 ± 0.5	6.2 ± 0.8	40	27 ± 1		
**37** ^d^	Bn	Bn	Cyclopropyl	NHCbz	CH_2_COOMe	3.0 ± 0.1	>100	11	3.6 ± 0.1		
**Subseries 4: modifications at R_5_**											
**38** ^e^	Bn	Bn	4-F-Bn	NHBoc	CH_3_	12 ± 1	15 ± 3	100	48 ± 2		
**39** ^d^	Bn	Bn	Bn	NHBoc	H	11 ± 0	15 ± 2	100	49 ± 1		
**40** ^e^	Bn	Bn	4-F-Bn	NHBoc	CH_2_CH_2_COOMe	4.8 ± 1.7	11 ± 4	100	50 ± 3	21	
**41** ^e^	Bn	Bn	4-F-Bn	NHBoc	CH_2_CONH_2_	54 ± 4	64 ± 8	>100	82 ± 13		
**42** ^d^	Bn	Bn	Bn	NHBoc	CH_2_CONH_2_	13 ± 0	16 ± 3	100	40 ± 6		
**43** ^e^	Bn	Bn	4-F-Bn	NHBoc	CH_2_COOH	>100	>100	>100	>100		
**44** ^d^	Bn	Bn	Bn	NHBoc	CH_2_COOH	>100	>100	>100	>100		
**45** ^e^	Bn	Bn	4-F-Bn	NHBoc	CH_2_Ph	0.85 ± 0.07	1.1 ± 0.2	50	4.4 ± 0.8		
**46** ^e^	Bn	Bn	Bn	NHBoc	CH_2_Ph	7.4 ± 1.3	4.5 ± 0.2	≥100	75 ± 0	≥14	17
**47** ^e^	Bn	Bn	Bn	NHBoc	CH_2_Indolyl	57 ± 17	49 ± 12	>100	>100		
**48** ^d^	Bn	Bn	Bn	NHBoc	CH_2_Ph-4-OH	11 ± 1	9.3 ± 0	>100	>100		>11
**49**	Bn	Bn	Bn	NHBoc	CH_2_Ph-4-F	10 ± 0	8.4 ± 0	≥100	>100	≥10	>12
**50**	Bn	Bn	Bn	NHBoc	CH_2_Ph-4-Cl	10 ± 0	9.7 ± 0.4	≥100	>100	≥10	≥10
**51**	Bn	Bn	Bn	NHBoc	CH_2_Ph-4-OMe	8.2 ± 1.9	4.8 ± 0.4	≥100	>100	≥12	≥21
**52**	Bn	Bn	Bn	NHBoc	CH_2_Ph-4-Me	6.6 ± 1.8	4.7 ± 0.6	≥100	>100	≥15	>21
**Subseries 5: combination of modifications at R_1_-R_5_**											
**53**	(CH_2_)_2_Ph	Bn	Bn	NHCbz	CH_2_COOMe	6.1 ± 2.0	5.8 ± 1.9	40	24 ± 2		
**54**	(CH_2_)_2_Ph-4-Me	Bn	Bn	NHCbz	CH_2_COOMe	10 ± 0	8.4 ± 0.5	40	24 ± 2		
**55**	(CH_2_)_2_Ph-4NO_2_	Bn	Bn	NHCbz	CH_2_COOMe	10 ± 0	7.7 ± 0.9	60	>100		>13
**56**	(CH_2_)_2_Ph-4-F	Bn	Bn	NHCbz	CH_2_COOMe	7.6 ± 1.8	6.0 ± 1.9	32	23 ± 3		
**57**	Bn	Bn	PhNH(CH_2_)_2_	NHCbz	CH_2_COOMe	10 ± 0	7.1 ± 0.9	40	51 ± 9		
**58**	(CH_2_)_2_Ph	Bn	PhNH(CH_2_)_2_	NHCbz	CH_2_COOMe	7.9 ± 1.9	5.4 ± 1.4	24	20 ± 4		
**59**	(CH_2_)_2_Ph	Bn	4-Me-Bn	NHCbz	CH_2_COOMe	8.8 ± 0.7	5.1 ± 1.0	16	15 ± 5		
**60**	4-F-Bn	Bn	Bn	NHBoc	CH_2_Ph	4.5 ± 0.2	4.0 ± 0.1	≥100	>100	≥22	>25
**61**	4-Cl-Bn	Bn	Bn	NHBoc	CH_2_Ph	36 ± 9	30 ± 7	>100	>100		
**62**	4-OMe-Bn	Bn	Bn	NHBoc	CH_2_Ph	5.9 ± 1.8	4.3 ± 0.4	40	28 ± 0		
**63**	4-NO_2_-Bn	Bn	Bn	NHBoc	CH_2_Ph	82 ± 15	77 ± 9	≥100	>100		
**Reference compounds**											
**GS-441524**						3.5 ± 0.0	2.3 ± 0.2	>100	>100	>29	>43
**K22** [36]						2.6 ± 0.5	3.6 ± 0.6	50	28 ± 3	19	

^a^ 50% effective concentration for protection against virus-induced CPE, as assessed by microscopic scoring and MTS cell viability assay. ^b^ MCC: minimal cytotoxic concentration causing microscopically visible alterations in cell morphology; CC_50_: 50% cytotoxic concentration based on MTS cell viability assay. ^c^ Ratio of MCC to EC_50_-microscopy or CC_50_ to EC_50_-MTS; only the selectivity index (SI) values of 10 or higher are shown. ^d^ See reference [9] for compound synthesis and analysis. ^e^ See reference [10] for compound synthesis and analysis.

**Table 2:** Anti-SARS-CoV-2 activity of selected 1,4,4-trisubstituted piperidine analogues.

Compound	Antiviral activity (µM)		Cytotoxicity ^b^ (µM)
	EC_90_^a^	EC_99_^a^	CC_50_
**33**	9.3	14	90
**34**	8.0	13	79
**52**	1.7	3.9	51
**60**	2.2	5.2	63
**GS-441524**	3.2	4.6	>16

^a^ Compound concentrations to achieve 10-fold (EC_90_) or 100-fold (EC_99_) reduction in the number of viral RNA copies in the supernatant at day 3 p.i., based on RT-qPCR analysis. The assay was performed in A549-AT cells. ^b^ CC_50_: 50% cytotoxic concentration determined by MTS cell viability assay in mock-infected A549-AT cells. Values based on nonlinear least-squares regression analysis of the results from three independent experiments, using GraphPad Prism software.

**Table 3:** Inhibitory activity on SARS-CoV-2 M^pro^ enzyme activity.

Compound	IC_50_ (µM)
**1**	161 ± 35
**33**	64 ± 8
**34**	38 ± 5
**45**	15 ± 2
**46**	22 ± 2
**52**	14 ± 4
**63**	196 ± 39
**60**	68 ± 21

## Data Availability

The data presented in this study are available in this study.
